# Peer victimization in early adolescence and maladjustment in adulthood

**DOI:** 10.1007/s00787-024-02532-5

**Published:** 2024-07-29

**Authors:** Tina Kretschmer, Rozemarijn van der Ploeg, Tessa Kaufman

**Affiliations:** 1 Faculty of Behavioural and Social Sciences, University of Groningen, Grote Rozenstraat 38, 9713TJ Groningen, The Netherlands; 2 Utrecht University, Utrecht, The Netherlands

**Keywords:** Bullying-victimization, Mental health, Longitudinal

## Abstract

Studies report different effect sizes for associations between peer victimization and later maladjustment. A possible origin of this heterogeneity is the length of the interval between victimization and maladjustment assessments. Effect sizes might also vary as a function of reporter. Longitudinal data from TRAILS, a study of adolescents followed from age 11 to 29 (baseline *n* = 2229) were used to test whether peer victimization assessed from adolescents themselves, their parents, teachers, and peers predicted internalizing symptoms, thought problems, and somatic complaints at six follow-ups with a temporal distance of up to 19 years. Neither self- nor peer-reported victimization predicted later maladjustment. In contrast, parent-reported victimization stably predicted adult maladjustment. Teacher-reported victimization also predicted maladjustment but associations were weaker and largely non-significant when parent reports were accounted for simultaneously. Parent-reported peer victimization has traditionally played a minor role in bullying research as parents are usually not present when victimization occurs. The results of this study however suggest that parents should be listened to when talking about their offspring being victimized.

**Supplementary Information:**

The online version contains supplementary material available at 10.1007/s00787-024-02532-5.

## Introduction

Peer victimization describes goal-directed and repeated negative behaviour towards an individual who finds it difficult to defend him- or herself and is experienced by approximately one in three young people [10]. Research suggests an increased risk among victimized individuals especially for internalizing problems such as social withdrawal and anxiety, psychotic symptoms such as intrusive thoughts and hallucination, and somatic symptoms such as headaches and sleep problems [6, 9, 15, 20, 23]. Various mechanisms have been proposed, including that being victimized is a significant source of stress that results in changes to physiological processes including cortisol activity [17] which has also been linked to depression and anxiety [32] and somatic complaints [19]. Peer victimization might also impair later mental health via cognitive processes such as rumination and lack of self-confidence that are, in turn, precursors of depressive symptoms [3]. Finally, it is also possible that peer victimization and mental health have a shared genetic basis that explains the overlap [29].

Effect sizes for associations between peer victimization and maladjustment symptoms vary considerably, however, and methodological choices including reporter and interval between exposure and outcome assessments might be sources of heterogeneity. Unfortunately, studies where associations between victimization and maladjustment are tested for different intervals and reporters are lacking. Moreover, longitudinal studies do not always account for baseline levels of maladjustment. We tackle these gaps in the present study.

### Interval between victimization and outcome

Although there are long-term longitudinal studies on links between peer victimization and maladjustment (e.g., [22]), most have assessed outcomes only a few years later than victimization. For instance, a recent meta-analysis on longitudinal studies reports intervals between exposure and outcome of not more than up to 5 years [5]. The maximum intervals between assessments of victimization and psychotic symptoms were ten and 15 years, respectively in meta-analyses, though most included studies assessed exposure and outcomes with much shorter time interval [6, 23]. A meta-analysis on victimization and sleep problems even relied solely on cross-sectional studies [25]. This is unfortunate as a long-term perspective helps to understand the persistence of peer victimization effects on maladjustment, especially across transitions from one developmental period to the next. Indeed, some associations detected when outcomes were assessed fairly close in time were not found when outcomes were assessed with a greater time difference [20]. This suggests that people might recover and put what has happened behind themselves once they have firmly moved into a different life stage. Here, we examine outcomes assessed on six occasions between ages 13–29 which allows for conclusions about the persistence of peer victimization effects.

### Reporter of victimization

Effect sizes for associations between victimization and maladjustment vary considerably and one source of heterogeneity might be “whom you ask”: Different reporters do not necessarily agree on who is victimized [14] and tend to identify different types of victims [18, 28]. Self-reported victims often have negative self-perceptions and feel excluded which might amplify maladjustment risk [28]. Reports from peers, usually obtained via nomination procedures, integrate perspectives of multiple people, sometimes the entire classroom, but most nomination studies exclude out-of-school peers and thus do not capture all possible victimization contexts. Teacher-identified victims are often highly visible and occupy a poor position in the peer group [28]. Finally, parents’ knowledge is dependent on what is shared with them rather than them being actively involved in victimization contexts. Further, adolescents’ and parents’ views can provide valuable reports of victimization chronicity, i.e., stable victimization across years and sometimes even contexts, compared to peers and teachers who are usually present for a shorter duration in adolescents’ lives. In short, reporters have unique viewpoints but few studies explicitly include associations between victimization and maladjustment for different reporters.

### Controlling for baseline

By far not all studies on peer victimization and later maladjustment have included baseline measures of maladjustment which hinders conclusions about the temporal order of effects. Take, for instance, withdrawal and anxiety as frequently studied internalizing “outcomes” of peer victimization: Not only are internalizing problems also predictors of peer problems [5], victimization and internalizing symptoms also share the same genetic basis, instead of victimization being a causal predictor of maladjustment [29]. Even though controlling for baseline maladjustment does not inform about causality to the extent that an experimental design would, regressing maladjustment symptoms on peer victimization while controlling for existing maladjustment symptoms can indicate worsening in symptoms as a function of victimization.

### Current study

We used longitudinal data with peer victimization assessed in early adolescence from adolescents themselves, their parents, teachers, and peers, and maladjustment symptoms assessed at ages 13, 16, 19, 22, 26, and 29. We focus on withdrawal and anxiety as facets of internalizing problems, thought problems as measure of psychotic symptoms and somatic complaints as umbrella measure for headaches, sleep problems, and similar symptoms. These are the most widely studied forms of maladjustment in the peer victimization literature yet for all of them we observe substantial heterogeneity in effect sizes that might be due to interval and reporter. We expected associations to be stronger when exposure and outcome were assessed with shorter interval.

We also explored associations between victimization and outcomes for different reporters. Based on prior work, we expected associations to be stronger for self-reported victimization than for other reporters. We did not have hypotheses pertaining to whether parents’ or teachers’ or peers’ reports of victimization would predict withdrawal, anxiety, thought problems, and somatic complaints more or less strongly. Additional analyses were conducted to examine whether patterns of results were stable to modifications of the model. In all analyses, we controlled for baseline maladjustment as well as sex, family socioeconomic status, and family structure, in line with earlier work on longitudinal outcomes of peer victimization.

## Methods

### Participants

Data come from the TRacking Adolescents' Individual Lives Survey (TRAILS), a cohort study of Dutch adolescents. Initially, all 135 primary schools in five municipalities the northern Netherlands were approached of which 122 agreed to participate. Through the schools, 2935 children were invited to participate of whom 2229 (51% female) did so at T1. Data collection at the first assessment wave took place in 2001 and 2002 (mean age 11.1 years), the second wave took place in 2003 and 2004 (mean age 13.6 years), the third wave in 2006 and 2007 (mean age 16.3 years), the fourth wave in 2008 to 2010 (mean age 19.1 years), the fifth wave was conducted in 2012 and 2013 (mean age 22.3 years), the sixth wave took place in 2016 (mean age 25.7 years) and the seventh wave was conducted in 2020 when participants were on average 28.9 years old. Here, we use data from all waves. The first wave approximately corresponds to the semi-final year of primary school and the second wave corresponds to the first year of secondary school. Ethics approval for TRAILS was obtained from the Dutch Central Committee on Research involving Human Subjects and all participants provided informed consent. Details about TRAILS have been published in several reports (e.g., [16]). Attrition information for the measures used in the present study is reported below.

### Measures

*Self-reported peer victimization* was assessed at age 11 using one item from the Youth Self Report [2] “I get bullied a lot”, rated as 0 = *not at all*, 1 = *a little bit/sometimes* 2 = *clearly*/*often*. 32% of the participants reported to be victimized sometimes (26%) or often (6%).

*Parent-reported peer victimization* was assessed when participants were 11 years old, using one item from the Child Behaviour Checklist [2] “My child gets teased a lot”, rated as 0 = *does not apply at all* 1 = *applies a little bit/sometimes* 2 = *applies often*. 31% of parents reported that their child had been teased sometimes (27%) or often (4%).

*Teacher-reported peer victimization* was assessed when participants were 11 years old using a Social Problems vignette based on the Teachers Report Form [1]. Teachers were provided with short vignettes including several traits and behaviours that correspond to items – and vignettes to subscales – in the Youth Self Report and Child Behaviour Checklist. The Social Problems vignette contained descriptions that match peer victimization items such as “is teased a lot”. For each TRAILS participant, teachers indicated how well the vignette described the child (0 = *does not apply at all* to 5 = *almost always applies)*. According to teachers, 34% of children experienced victimization (5% often).

*Peer-reported peer victimization* was assessed from a subsample of TRAILS participants who took part in classroom-based assessments at age 11, in which TRAILS participants and their classmates nominated each other on a range of domains including “who is being bullied” [7, 27]. Peer nominations were only collected in classrooms with at least ten TRAILS-respondents. Schools that had agreed to participate provided the names of classmates of TRAILS respondents. Just under half of all respondents (*n* = 1065) participated in the peer nomination procedure. Note that nominations included the entire classroom, not only TRAILS participants. More detail on the peer nominations can be found elsewhere [7, 12]. The nominations received for being victimized were divided by the total number of participating pupils in the class, that is, the maximum number of nominations possible. These proportion scores take class size into account and range from 0 to 1, with higher scores indicating more victimization nominations.

*Baseline maladjustment* was assessed at age 11 using the Youth Self Report [2] withdrawal, anxiety, thought problems, and somatic complaints subscales. Withdrawal consists of eight items including “I try to have little to do with others” (*α* = 0.64). Anxiety consists of 13 items including “I’m scared of school” (*α* = 0.78). Thought problems consists of 12 items including “I have thoughts that others find strange” (*α* = 0.72). Somatic complaints consists of 10 items including “I feel tired for no reason” (*α* = 0.75). All items were rated on a scale from 0 = *not at all* to 2 = *clearly or often*. Scores were averaged for each subscale with higher scores reflecting more symptoms.

*Maladjustment outcomes* were assessed at ages 13 and 16 using the Youth Self Report [2], including withdrawal, anxiety, thought problems, and somatic complaints subscales as described for baseline maladjustment. Internal consistency was satisfactory with *α*’s ranging from 0.67 (thought problems at age 16) to 0.84 (somatic complaints at age 13). From age 19 onward, the Adult Self Report [2] withdrawal, anxiety, thought problems, and somatic complaints subscales were assessed. In the adult version of the questionnaire, withdrawal consists of nine items (*α*’s ranging from = 0.76 to 0.80), anxiety consists of 18 items (*α*’s ranging from = 0.91 to 0.93), thought problems consists of nine items (*α*’s ranging from = 0.65 to 0.69), and somatic complaints consists of 12 items (*α*’s ranging from = 0.75 to 0.82). Again, items were rated on a scale from 0 = *not at all* to 2 = *clearly/often*, higher scores reflect more maladjustment.

*Covariates* *Family socioeconomic status* was constructed from mothers’ and fathers’ educational and occupational levels and family income as measured at TRAILS T1 (~ age 11). Educational level of parents was coded in five categories based on the International Standard Classification of Occupations [8]. Disposable family income was measured on a scale ranging from less than €680 (1) to more than €3857 (9). Family socioeconomic status was consequently operationalized as the average of the standardized five items (*α* = 0.84, *M* = -0.05, *SD* = 0.80, *range* = – 1.94 to 1.73); this indicator is commonly used in TRAILS analyses [26]. Participant *sex* was coded as 0 = *girl*, 1 = *boy*, 50.7% of participants were female at T1. *Family structure* reflects whether TRAILS participants lived with both biological parents at T1 (69.7%).

### Attrition analyses

Individuals were more likely to have participated in age 13 to 29 assessments if they came from higher SES families (*t* ranging from 3.62 to 12.92, *p* < 0.001), were female (*χ*^*2*^ ranging from 4.03 to 124.58, *p* < 0.001), and lived with both biological parents present in the home at age 11 (*χ*^*2*^ ranging from 7.26 to 38.80, *p* < 0.001). Participants at the age 13 assessment did not differ by victimization status, participation at the age 16 assessment was more likely for those whose parents (*t*(2026) = 2.35, *p* = 0.02) and teachers (*t*(1904) = 2.70, *p* = 0.007) had not indicated victimization at the age 11 assessment, participation at the age 19 assessment was more likely for those whose teachers (*t*(1885) = 3.86, *p* < 0.001) and peers (*t*(1044) = 1.97, *p* = 0.05) had not indicated victimization, participation at the age 22 assessment was more likely for those whose teachers (*t*(1921) = 3.80, *p* < 0.001) had not indicated victimization, participation at the age 26 assessment was more likely for those whose parents (*t*(2047) = 2.10, *p* = 0.04) and teachers (*t*(1923) = 3.92, *p* < 0.001) had not indicated victimization, and participation at the age 29 assessment was more likely for those whose teachers (*t*(1923) = 3.89, *p* < 0.001) had not indicated victimization. All effect sizes were modest and none of the other victimization reports were associated with follow-up participation. We used full information maximum likelihood estimation so all analyses are based on a sample of *n* = 2229 but Table 1 indicates exact sample sizes per measure.

**Table 1:** Descriptive statistics for measures used in main analyses

	*n*	Mean	SD	Range
Predictors				
Peer victimization at age 11				
Self-report	2185	0.38	0.59	0.00–2.00
Parent-report	2049	0.35	0.59	0.00–2.00
Teacher-report	1925	0.58	0.96	0.00–4.00
Peer-report	1064	0.04	0.08	0.00–0.63
Outcomes				
Maladjustment at age 13				
Withdrawal	2090	0.34	0.30	0.00–1.88
Anxiety	2092	0.31	0.29	0.00–1.92
Thought problems	2086	0.23	0.24	0.00–1.58
Somatic complaints	2075	0.33	0.29	0.00–1.70
Maladjustment at age 16				
Withdrawal	1658	0.38	0.32	0.00–1.75
Anxiety	1659	0.29	0.29	0.00–2.00
Thought problems	1659	0.23	0.22	0.00–1.50
Somatic complaints	1644	0.30	0.28	0.00–1.50
Maladjustment at age 19				
Withdrawal	1695	0.23	0.27	0.00–1.67
Anxiety	1693	0.32	0.33	0.00–1.89
Thought problems	1657	0.24	0.22	0.00–1.50
Somatic complaints	1690	0.17	0.25	0.00–1.92
Maladjustment at age 22				
Withdrawal	1498	0.23	0.29	0.00–1.67
Anxiety	1498	0.31	0.34	0.00–1.88
Thought problems	1498	0.22	0.21	0.00–1.40
Somatic complaints	1498	0.25	0.25	0.00–1.67
Maladjustment at age 26				
Withdrawal	1315	0.28	0.31	0.00–1.78
Anxiety	1315	0.39	0.38	0.00–1.94
Thought problems	1315	0.23	0.22	0.00–1.40
Somatic complaints	1315	0.29	0.28	0.00–1.67
Maladjustment at age 29				
Withdrawal	1118	0.25	0.29	0.00–1.67
Anxiety	1118	0.36	0.37	0.00–1.78
Thought problems	1118	0.20	0.21	0.00–1.20
Somatic complaints	1118	0.29	0.29	0.00–1.83
Covariates				
Maladjustment at age 11				
Withdrawal	2186	0.34	0.29	0.00–1.50
Anxiety	2194	0.32	0.27	0.00–1.54
Thought problems	2185	0.28	0.26	0.00–1.75
Somatic complaints	2175	0.43	0.31	0.00–1.60
Family SES at age 11	2187	-0.05	0.80	– 1.94 to 1.73
Sex		1131 (50.7%) female; 1098 (49.3%) male		
Family structure at age 11		1553 (69.7%) with both biological parents; 676 (30.3%) not with both biological parents		

### Analytic strategy

#### Main analyses

We computed regression models in Stata 17 in which withdrawal, anxiety, thought problems, and somatic complaints at ages 13 through 29 were predicted by peer victimization at age 11, controlling for family SES and structure, sex, and baseline withdrawal, anxiety, thought problems, and somatic complaints. We employed the SEM command to be able to use full information maximum likelihood estimation to deal with missing values, and report standardized coefficients and *p*-values as well as amount of variance explained. Next to separate models for each reporter, we also computed models in which all reporters were included simultaneously. Following these main analyses, we conducted a series of additional tests to probe the stability of the pattern of result.

#### Additional analyses

First, we computed more severe victimization indices by coding only those individuals as being victimized who themselves, their parents or teacher had indicated that they were victimized “often” or who obtained within the top 10% of nominations by peers. Second, we examined associations between age 13 victimization and later withdrawal, anxiety, thought problems, and somatic complaints, i.e., with outcomes assessed at ages 16, 19, 22, 26, and 29 (information on measures and attrition is provided in Supplementary Table 1). Third, we explored whether associations with outcomes would look different for chronic victimization. To this end, we computed scores representing any victimization both at ages 11 and 13 versus no or only temporary victimization. Fourth, although withdrawal, anxiety, thought problems, and somatic complaints represent different facets of internalizing problems and are usually examined separately as correlates of peer victimization, they also show considerable overlap. For this reason, we computed additional analyses where internalizing problems were conceptualized as latent variable. For all additional analyses, separate models per reporter were computed first, followed by models with all reporters included and we controlled for family SES and structure, sex, and baseline maladjustment throughout.

## Results

### Main analyses: Peer victimization at age 11 and maladjustment at ages 13 through 29

Descriptive statistics for the variables included in the main analyses are provided in Table 1 and pairwise correlations are depicted in a heatmap in *Supplementary Fig. 1*. Correlations between reporters were modest to moderate, supporting separate analyses instead of using a composite of the different reports. Correlations between self-, parent- and teacher-reported victimization and maladjustment symptoms were modest but statistically significant for all assessments whereas correlations between peer-reported victimization and maladjustment were significantly related to maladjustment in early adolescence but not later. There was substantial overlap between symptoms as evident from strong correlations for symptoms assessed concurrently. Decreasing trends as assessments were further apart indicate moderate stability.

Table 2a–f depict associations between victimization at age 11 and withdrawal, anxiety, thought problems, and somatic complaints assessed at ages 13, 16, 19, 22, 26, and 29, controlling for sex, family SES and structure, and baseline withdrawal, anxiety, thought problems, and somatic complaints. Parent- and teacher-reported victimization were linked to all forms of maladjustment at all ages with only two exceptions: teacher-reported victimization did not predict thought problems at age 13 nor somatic complaints at age 16. Effect sizes were somewhat bigger for parent-reported victimization as predictor of maladjustment symptoms in late adolescence and again at age 26 but no clear in- or decrease in effect size over time can be observed. Self-reported victimization was associated with maladjustment symptoms occurring temporary closer to the victimization experiences but associations decreased in size and became non-significant for adult measurement waves. Finally, peer-reported victimization was not associated with any form of maladjustment except for anxiety at age 13. When all reporters were entered into the model simultaneously, only parent-reported victimization remained a significant predictor of all forms of maladjustment except anxiety and thought problems at age 29. Associations between self-reported victimization and maladjustment symptoms vanished as did more than half of all associations between teacher-reported victimization and maladjustment symptoms.

**Table 2:** (a) Associations between peer victimization at age 11 and maladjustment at age 13, (b) associations between peer victimization at age 11 and maladjustment at age 16, (c) associations between peer victimization at age 11 and maladjustment at age 19, (d) associations between peer victimization at age 11 and maladjustment at age 22, (e) associations between peer victimization at age 11 and maladjustment at age 26, (f) associations between peer victimization at age 11 and maladjustment at age 29

(a)												
	Withdrawal			Anxiety			Thought problems			Somatic complaints		
	*ß*	*p*	*R*^*2*^	*ß*	*p*	*R*^*2*^	*ß*	*p*	*R*^*2*^	*ß*	*p*	*R*^*2*^
Separate models per reporter												
SR peer victimization	**0.05**	**0.01**	0.21	**0.10**	** < 0.001**	0.27	0.03	0.11	0.18	**0.05**	**0.01**	0.23
Sex	**– 0.14**	** < 0.001**		**– 0.22**	** < 0.001**		**– 0.12**	** < 0.001**		**– 0.20**	** < 0.001**	
Family SES	0.003	0.88		0.03	0.12		0.03	0.18		**– 0.08**	** < 0.001**	
Family structure	**0.04**	**0.04**		0.01	0.53		**0.07**	**0.001**		0.01	0.79	
Baseline maladjustment	**0.40**	** < 0.001**		**0.40**	** < 0.001**		**0.39**	** < 0.001**		**0.39**	** < 0.001**	
PR peer victimization	**0.14**	** < 0.001**	0.22	**0.12**	** < 0.001**	0.27	**0.08**	** < 0.001**	0.19	**0.08**	** < 0.001**	0.23
Sex	**– 0.15**	** < 0.001**		**– 0.23**	** < 0.001**		**– 0.13**	** < 0.001**		**– 0.21**	** < 0.001**	
Family SES	0.02	0.46		0.03	0.08		0.04	0.10		**– 0.07**	** < 0.001**	
Family structure	0.04	0.07		0.01	0.65		**0.06**	**0.002**		0.003	0.89	
Baseline maladjustment	**0.39**	** < 0.001**		**0.41**	** < 0.001**		**0.39**	** < 0.001**		**0.40**	** < 0.001**	
TR peer victimization	**0.08**	** < 0.001**	0.21	**0.05**	**0.03**	0.26	0.03	0.12	0.18	**0.04**	**0.04**	0.23
Sex	**– 0.15**	** < 0.001**		**– 0.22**	** < 0.001**		**– 0.12**	** < 0.001**		**– 0.20**	** < 0.001**	
Family SES	0.01	0.62		0.03	0.20		0.03	0.17		**– 0.08**	** < 0.001**	
Family structure	0.04	0.05		0.01	0.53		**0.07**	**0.001**		0.01	0.79	
Baseline maladjustment	**0.41**	** < 0.001**		**0.43**	** < 0.001**		**0.39**	** < 0.001**		**0.40**	** < 0.001**	
PeerR peer victimization	0.02	0.46	0.20	**0.06**	**0.04**	0.26	0.05	0.12	0.18	0.06	0.07	0.23
Sex	**– 0.14**	** < 0.001**		**– 0.22**	** < 0.001**		**– 0.12**	** < 0.001**		**– 0.20**	** < 0.001**	
Family SES	– 0.001	0.97		0.02	0.23		0.03	0.18		**– 0.08**	** < 0.001**	
Family structure	**0.04**	**0.04**		0.01	0.53		**0.07**	**0.001**		0.01	0.79	
Baseline maladjustment	**0.42**	** < 0.001**		**0.44**	** < 0.001**		**0.39**	** < 0.001**		**0.40**	** < 0.001**	
Model with all reporters included simultaneously												
SR peer victimization	– 0.003	0.90	0.22	**0.06**	**0.01**	0.28	– 0.01	0.85	0.19	0.02	0.48	0.23
PR peer victimization	**0.12**	** < 0.001**		**0.09**	** < 0.001**		**0.07**	**0.003**		**0.06**	**0.01**	
TR peer victimization	0.05	0.05		– 0.002	0.93		– 0.001	0.97		0.001	0.96	
PeerR peer victimization	– 0.01	0.86		0.03	0.32		0.04	0.27		0.04	0.22	
Sex	**– 0.15**	** < 0.001**		**– 0.23**	** < 0.001**		**– 0.13**	** < 0.001**		**– 0.21**	** < 0.001**	
Family SES	0.02	0.32		**0.04**	**0.04**		0.04	0.08		**– 0.07**	**0.001**	
Family structure	0.04	0.08		0.01	0.70		**0.06**	**0.002**		0.001	0.95	
Baseline maladjustment	**0.39**	** < 0.001**		**0.40**	** < 0.001**		**0.39**	** < 0.001**		**0.39**	** < 0.001**	

(b)												
	Withdrawal			Anxiety			Thought problems			Somatic complaints		
	*ß*	*p*	*R*^*2*^	*ß*	*p*	*R*^*2*^	*ß*	*p*	*R*^*2*^	*ß*	*p*	*R*^*2*^
Separate models per reporter												
SR peer victimization	0.05	0.04	0.13	0.03	0.17	0.21	**0.06**	**0.02**	0.12	**0.05**	**0.02**	0.21
Sex	**– 0.12**	** < 0.001**		**– 0.28**	** < 0.001**		**– 0.08**	** < 0.001**		**– 0.31**	** < 0.001**	
Family SES	**– 0.06**	**0.02**		– 0.01	0.68		– 0.03	0.29		**– 0.07**	**0.002**	
Family structure	**0.05**	**0.03**		0.03	0.26		**0.10**	** < 0.001**		0.04	0.07	
Baseline maladjustment	**0.30**	** < 0.001**		**0.32**	** < 0.001**		**0.29**	** < 0.001**		**0.27**	** < 0.001**	
PR peer victimization	**0.11**	** < 0.001**	0.14	**0.09**	** < 0.001**	0.22	**0.10**	** < 0.001**	0.13	**0.09**	** < 0.001**	0.21
Sex	**– 0.12**	** < 0.001**		**– 0.29**	** < 0.001**		**– 0.09**	** < 0.001**		**– 0.31**	** < 0.001**	
Family SES	– 0.04	0.07		0.001	0.96		– 0.02	0.47		**– 0.07**	**0.004**	
Family structure	0.05	0.05		0.02	0.34		**0.10**	** < 0.001**		0.04	0.09	
Baseline maladjustment	**0.30**	** < 0.001**		**0.32**	** < 0.001**		**0.30**	** < 0.001**		**0.28**	** < 0.001**	
TR peer victimization	**0.06**	**0.03**	0.13	**0.06**	**0.02**	0.22	**0.07**	**0.01**	0.12	0.04	0.07	0.21
Sex	**– 0.12**	** < 0.001**		**– 0.28**	** < 0.001**		**– 0.09**	** < 0.001**		**– 0.31**	** < 0.001**	
Family SES	**– 0.05**	**0.03**		– 0.004	0.85		– 0.02	0.34		**– 0.08**	**0.001**	
Family structure	**0.05**	**0.04**		0.02	0.29		**0.10**	** < 0.001**		0.04	0.07	
Baseline maladjustment	**0.31**	** < 0.001**		**0.33**	** < 0.001**		**0.30**	** < 0.001**		**0.28**	** < 0.001**	
PeerR peer victimization	0.02	0.53	0.13	– 0.03	0.46	0.21	– 0.05	0.12	0.12	0.03	0.34	0.21
Sex	**– 0.12**	** < 0.001**		**– 0.28**	** < 0.001**		**– 0.08**	**0.001**		**– 0.31**	** < 0.001**	
Family SES	**– 0.06**	**0.01**		– 0.02	0.49		– 0.04	0.12		**– 0.08**	**0.001**	
Family structure	**0.05**	**0.03**		0.03	0.21		**0.10**	** < 0.001**		0.04	0.06	
Baseline maladjustment	**0.32**	** < 0.001**		**0.33**	** < 0.001**		**0.31**	** < 0.001**		**0.28**	** < 0.001**	
Model with all reporters included simultaneously												
SR peer victimization	0.01	0.87	0.14	– 0.003	0.92	0.22	0.03	0.28	0.14	0.01	0.62	0.21
PR peer victimization	**0.11**	** < 0.001**		**0.09**	** < 0.001**		**0.09**	**0.001**		**0.08**	**0.004**	
TR peer victimization	0.02	0.55		0.03	0.24		0.05	0.08		0.003	0.93	
PeerR peer victimization	– 0.01	0.90		– 0.03	0.36		**– 0.08**	**0.04**		0.03	0.42	
Sex	**– 0.12**	** < 0.001**		**– 0.29**	** < 0.001**		**– 0.09**	** < 0.001**		**– 0.31**	** < 0.001**	
Family SES	– 0.04	0.09		0.003	0.91		– 0.02	0.54		**– 0.06**	**0.01**	
Family structure	0.05	0.06		0.02	0.35		**0.09**	** < 0.001**		0.04	0.11	
Baseline maladjustment	**0.30**	** < 0.001**		**0.32**	** < 0.001**		**0.29**	** < 0.001**		**0.28**	** < 0.001**	

(c)												
	Withdrawal			Anxiety			Thought problems			Somatic complaints		
	*ß*	*p*	*R*^*2*^	*ß*	*p*	*R*^*2*^	*ß*	*p*	*R*^*2*^	*ß*	*p*	*R*^*2*^
Separate models per reporter												
SR peer victimization	0.03	0.20	0.09	**0.07**	**0.004**	0.15	**0.09**	**0.001**	0.10	**0.07**	**0.003**	0.11
Sex	– 0.002	0.92		**– 0.16**	** < 0.001**		– 0.02	0.41		**– 0.22**	** < 0.001**	
Family SES	– 0.05	0.05		– 0.01	0.72		– 0.03	0.33		**– 0.07**	**0.003**	
Family structure	**0.09**	** < 0.001**		**0.10**	** < 0.001**		**0.12**	** < 0.001**		**0.07**	**0.01**	
Baseline maladjustment	**0.25**	** < 0.001**		**0.29**	** < 0.001**		**0.23**	** < 0.001**		**0.17**	** < 0.001**	
PR peer victimization	**0.16**	** < 0.001**	0.11	**0.15**	** < 0.001**	0.17	**0.16**	** < 0.001**	0.11	**0.13**	** < 0.001**	0.12
Sex	– 0.01	0.58		**– 0.17**	** < 0.001**		– 0.03	0.26		**– 0.23**	** < 0.001**	
Family SES	– 0.03	0.24		0.01	0.83		– 0.01	0.60		**– 0.06**	**0.01**	
Family structure	**0.08**	**0.001**		**0.09**	** < 0.001**		**0.11**	** < 0.001**		**0.06**	**0.01**	
Baseline maladjustment	**0.23**	** < 0.001**		**0.29**	** < 0.001**		**0.24**	** < 0.001**		**0.18**	** < 0.001**	
TR peer victimization	**0.12**	** < 0.001**	0.10	**0.12**	** < 0.001**	0.16	**0.12**	** < 0.001**	0.10	**0.15**	** < 0.001**	0.13
Sex	– 0.01	0.57		**– 0.17**	** < 0.001**		– 0.03	0.23		**– 0.23**	** < 0.001**	
Family SES	– 0.03	0.18		0.001	0.97		– 0.02	0.50		**– 0.06**	**0.02**	
Family structure	**0.09**	** < 0.001**		**0.09**	** < 0.001**		**0.11**	** < 0.001**		**0.06**	**0.01**	
Baseline maladjustment	**0.24**	** < 0.001**		**0.30**	** < 0.001**		**0.24**	** < 0.001**		**0.18**	** < 0.001**	
PeerR peer victimization	– 0.02	0.63	0.09	< 0.001	0.99	0.15	– 0.01	0.75	0.09	– 0.03	0.48	0.11
Sex	< 0.001	0.99		**– 0.15**	** < 0.001**		– 0.02	0.54		**– 0.22**	** < 0.001**	
Family SES	**– 0.05**	**0.03**		– 0.02	0.49		– 0.04	0.15		**– 0.09**	**0.001**	
Family structure	**0.10**	** < 0.001**		**0.10**	** < 0.001**		**0.12**	** < 0.001**		**0.07**	**0.003**	
Baseline maladjustment	**0.26**	** < 0.001**		**0.32**	** < 0.001**		**0.26**	** < 0.001**		**0.19**	** < 0.001**	
Model with all reporters included simultaneously												
SR peer victimization	– 0.03	0.26	0.12	0.01	0.64	0.18	0.03	0.38	0.12	0.01	0.61	0.14
PR peer victimization	**0.16**	** < 0.001**		**0.14**	** < 0.001**		**0.13**	** < 0.001**		**0.10**	**0.001**	
TR peer victimization	**0.10**	**0.001**		**0.09**	**0.001**		**0.09**	**0.003**		**0.14**	** < 0.001**	
PeerR peer victimization	– 0.06	0.14		– 0.05	0.21		– 0.06	0.150		– 0.08	**0.04**	
Sex	– 0.02	0.46		**– 0.17**	** < 0.001**		– 0.03	0.180		– 0.24	** < 0.001**	
Family SES	– 0.03	0.32		0.01	0.59		– 0.01	0.83		– 0.05	**0.04**	
Family structure	**0.08**	**0.001**		**0.08**	** < 0.001**		**0.11**	** < 0.001**		0.06	**0.02**	
Baseline maladjustment	**0.24**	** < 0.001**		**0.28**	** < 0.001**		**0.23**	** < 0.001**		0.18	** < 0.001**	

(d)												
	Withdrawal			Anxiety			Thought problems			Somatic complaints		
	*ß*	*p*	*R*^*2*^	*ß*	*p*	*R*^*2*^	*ß*	*p*	*R*^*2*^	*ß*	*p*	*R*^*2*^
Separate models per reporter												
SR peer victimization	0.02	0.42	0.06	0.05	0.06	0.10	0.03	0.24	0.06	**0.06**	**0.02**	0.15
Sex	0.01	0.78		**– 0.16**	** < 0.001**		– 0.03	0.19		**– 0.29**	** < 0.001**	
Family SES	– 0.05	0.09		00.02	0.51		**– 0.06**	**0.04**		**– 0.07**	**0.01**	
Family structure	0.05	0.06		**0.06**	**0.02**		**0.08**	**0.004**		0.04	0.10	
Baseline maladjustment	0.22	** < 0.001**		**0.23**	** < 0.001**		**0.20**	** < 0.001**		**0.18**	** < 0.001**	
PR peer victimization	**0.11**	** < 0.001**	0.08	**0.10**	** < 0.001**	0.11	**0.11**	** < 0.001**	0.07	**0.11**	** < 0.001**	0.16
Sex	– 0.01	0.98		**– 0.16**	** < 0.001**		– 0.04	0.11		**– 0.30**	** < 0.001**	
Family SES	– 0.03	0.26		0.03	0.33		– 0.04	0.11		**– 0.06**	**0.03**	
Family structure	0.05	0.09		**0.06**	**0.03**		**0.07**	**0.01**		0.04	0.14	
Baseline maladjustment	**0.21**	** < 0.001**		**0.23**	** < 0.001**		**0.20**	** < 0.001**		**0.19**	** < 0.001**	
TR peer victimization	**0.07**	**0.01**	0.06	**0.06**	**0.03**	0.10	**0.07**	**0.02**	0.06	**0.11**	** < 0.001**	0.15
Sex	– 0.01	0.99		**– 0.16**	** < 0.001**		– 0.04	0.12		**– 0.30**	** < 0.001**	
Family SES	– 0.04	0.19		0.02	0.42		– 0.05	0.08		**– 0.06**	**0.03**	
Family structure	0.05	0.07		**0.06**	**0.02**		**0.08**	**0.004**		0.04	0.12	
Baseline maladjustment	**0.22**	** < 0.001**		**0.24**	** < 0.001**		**0.20**	** < 0.001**		**0.19**	** < 0.001**	
PeerR peer victimization	– 0.03	0.43	0.06	– 0.01	0.98	0.10	0.02	0.59	0.06	– 0.01	0.98	0.14
Sex	0.01	0.68		**– 0.15**	** < 0.001**		– 0.03	0.20		**– 0.29**	** < 0.001**	
Family SES	– 0.05	0.06		0.01	0.70		**– 0.06**	**0.03**		**– 0.08**	**0.004**	
Family structure	0.05	0.05		**0.06**	**0.02**		**0.08**	**0.003**		0.05	0.07	
Baseline maladjustment	**0.23**	** < 0.001**		**0.25**	** < 0.001**		**0.21**	** < 0.001**		**0.19**	** < 0.001**	
Model with all reporters included simultaneously												
SR peer victimization	– 0.02	0.55	0.08	0.02	0.60	0.11	– 0.02	0.51	0.07	0.01	0.76	0.16
PR peer victimization	**0.12**	** < 0.001**		**0.09**	**0.003**		**0.10**	**0.001**		**0.10**	**0.001**	
TR peer victimization	0.06	0.07		0.04	0.22		0.05	0.16		**0.09**	**0.01**	
PeerR peer victimization	– 0.07	0.11		– 0.04	0.37		– 0.01	0.80		– 0.06	0.15	
Sex	– 0.002	0.92		**– 0.17**	** < 0.001**		– 0.04	0.09		**– 0.30**	** < 0.001**	
Family SES	– 0.03	0.28		0.03	0.26		– 0.04	0.16		– 0.05	0.06	
Family structure	0.04	0.10		**0.06**	**0.03**		**0.07**	**0.01**		0.04	0.16	
Baseline maladjustment	**0.21**	** < 0.001**		**0.22**	** < 0.001**		**0.20**	** < 0.001**		**0.19**	** < 0.001**	

(e)												
	Withdrawal			Anxiety			Thought problems			Somatic complaints		
	*ß*	*p*	*R*^*2*^	*ß*	*p*	*R*^*2*^	*ß*	*p*	*R*^*2*^	*ß*	*p*	*R*^*2*^
Separate models per reporter												
SR peer victimization	0.05	0.13	0.08	0.03	0.26	0.09	0.04	0.17	0.07	**0.06**	**0.04**	0.11
Sex	0.05	0.06		**– 0.11**	** < 0.001**		0.02	0.52		**– 0.25**	** < 0.001**	
Family SES	**– 0.06**	**0.04**		0.03	0.23		– 0.03	0.39		**– 0.07**	**0.01**	
Family structure	**0.10**	** < 0.001**		**0.08**	**0.01**		**0.12**	** < 0.001**		0.03	0.33	
Baseline maladjustment	**0.22**	** < 0.001**		**0.24**	** < 0.001**		**0.20**	** < 0.001**		**0.14**	** < 0.001**	
PR peer victimization	**0.17**	** < 0.001**	0.11	**0.14**	** < 0.001**	0.11	**0.13**	** < 0.001**	0.08	**0.15**	** < 0.001**	0.12
Sex	0.04	0.14		**– 0.13**	** < 0.001**		0.01	0.72		**– 0.26**	** < 0.001**	
Family SES	– 0.04	0.18		0.05	0.07		– 0.01	0.73		– 0.06	0.05	
Family structure	**0.09**	**0.002**		**0.06**	**0.02**		**0.11**	** < 0.001**		0.02	0.57	
Baseline maladjustment	**0.20**	** < 0.001**		**0.22**	** < 0.001**		**0.19**	** < 0.001**		**0.15**	** < 0.001**	
TR peer victimization	**0.12**	** < 0.001**	0.09	**0.10**	**0.002**	0.10	**0.11**	** < 0.001**	0.08	**0.13**	** < 0.001**	0.12
Sex	0.04	0.16		**– 0.12**	** < 0.001**		0.01	0.81		**– 0.26**	** < 0.001**	
Family SES	– 0.04	0.12		0.05	0.10		– 0.01	0.73		**– 0.06**	**0.04**	
Family structure	**0.10**	**0.001**		**0.07**	**0.01**		**0.11**	** < 0.001**		0.02	0.42	
Baseline maladjustment	**0.21**	** < 0.001**		**0.24**	** < 0.001**		**0.19**	** < 0.001**		**0.15**	** < 0.001**	
PeerR peer victimization	– 0.02	0.68	0.08	0.03	0.53	0.09	0.02	0.66	0.07	0.03	0.41	0.10
Sex	**0.06**	**0.04**		**– 0.11**	** < 0.001**		0.02	0.49		**– 0.25**	** < 0.001**	
Family SES	**– 0.07**	**0.02**		0.03	0.26		– 0.03	0.31		**– 0.08**	**0.01**	
Family structure	**0.10**	** < 0.001**		**0.08**	**0.01**		**0.12**	** < 0.001**		0.03	0.28	
Baseline maladjustment	**0.23**	** < 0.001**		**0.25**	** < 0.001**		**0.21**	** < 0.001**		**0.15**	** < 0.001**	
Model with all reporters included simultaneously												
SR peer victimization	– 0.03	0.36	0.12	– 0.03	0.33	0.11	– 0.03	0.43	0.08	– 0.02	0.50	0.13
PR peer victimization	**0.18**	** < 0.001**		**0.13**	** < 0.001**		**0.11**	**0.001**		**0.13**	** < 0.001**	
TR peer victimization	**0.10**	**0.01**		**0.07**	**0.04**		**0.09**	**0.02**		**0.08**	**0.03**	
PeerR peer victimization	– 0.07	0.10		– 0.01	0.78		– 0.02	0.65		0.01	0.89	
Sex	0.04	0.19		**– 0.13**	** < 0.001**		0.003	0.90		**– 0.27**	** < 0.001**	
Family SES	– 0.03	0.24		0.06	0.05		– 0.002	0.95		– 0.05	0.10	
Family structure	**0.09**	**0.002**		**0.06**	**0.03**		**0.11**	** < 0.001**		0.02	0.58	
Baseline maladjustment	**0.20**	** < 0.001**		**0.23**	** < 0.001**		**0.19**	** < 0.001**		**0.15**	** < 0.001**	

(f)												
	Withdrawal			Anxiety			Thought problems			Somatic complaints		
	*ß*	*p*	*R*^*2*^	*ß*	*p*	*R*^*2*^	*ß*	*p*	*R*^*2*^	*ß*	*p*	*R*^*2*^
Separate models per reporter												
SR peer victimization	0.04	0.19	0.07	0.04	0.22	0.08	0.04	0.17	0.06	0.04	0.23	0.09
Sex	0.04	0.14		**– 0.11**	** < 0.001**		0.01	0.87		**– 0.21**	** < 0.001**	
Family SES	**– 0.06**	**0.04**		0.01	0.86		– 0.05	0.13		**– 0.07**	**0.02**	
Family structure	**0.08**	**0.01**		**0.08**	**0.01**		**0.07**	**0.03**		0.05	0.11	
Baseline maladjustment	**0.21**	** < 0.001**		**0.22**	** < 0.001**		**0.19**	** < 0.001**		**0.16**	** < 0.001**	
PR peer victimization	**0.10**	**0.002**	0.08	**0.07**	**0.02**	0.08	**0.07**	**0.02**	0.06	**0.10**	**0.001**	0.10
Sex	0.04	0.20		**– 0.11**	** < 0.001**		0.01	0.95		**– 0.22**	** < 0.001**	
Family SES	– 0.05	0.11		0.01	0.69		– 0.04	0.20		– 0.06	0.06	
Family structure	**0.07**	**0.02**		**0.07**	**0.02**		0.06	0.05		0.04	0.18	
Baseline maladjustment	**0.20**	** < 0.001**		**0.22**	** < 0.001**		**0.20**	** < 0.001**		**0.16**	** < 0.001**	
TR peer victimization	**0.11**	**0.001**	0.08	**0.08**	**0.02**	0.08	**0.08**	**0.03**	0.06	**0.09**	**0.01**	0.10
Sex	0.03	0.25		**– 0.11**	** < 0.001**		– 0.01	0.99		**– 0.22**	** < 0.001**	
Family SES	– 0.05	0.11		0.01	0.69		– 0.04	0.19		– 0.06	0.05	
Family structure	**0.08**	**0.02**		**0.08**	**0.02**		**0.06**	**0.04**		0.05	0.14	
Baseline maladjustment	**0.21**	** < 0.001**		**0.22**	** < 0.001**		**0.19**	** < 0.001**		**0.16**	** < 0.001**	
PeerR peer victimization	0.03	0.53	0.07	0.01	0.76	0.08	– 0.01	0.89	0.05	0.07	0.13	0.09
Sex	0.04	0.13		**– 0.10**	**0.001**		0.01	0.77		**– 0.21**	** < 0.001**	
Family SES	**– 0.06**	**0.04**		0.01	0.96		– 0.05	0.09		**– 0.07**	**0.03**	
Family structure	**0.08**	**0.01**		**0.08**	**0.01**		**0.07**	**0.02**		0.05	0.10	
Baseline maladjustment	**0.22**	** < 0.001**		**0.23**	** < 0.001**		**0.21**	** < 0.001**		**0.16**	** < 0.001**	
Model with all reporters included simultaneously												
SR peer victimization	– 0.02	0.67	0.09	– < 0.001	0.99	0.09	0.01	0.85	0.06	– 0.03	0.36	0.10
PR peer victimization	**0.10**	**0.01**		0.06	0.08		0.07	0.06		**0.10**	**0.01**	
TR peer victimization	**0.09**	**0.02**		0.06	0.14		0.06	0.16		0.05	0.20	
PeerR peer victimization	– 0.02	0.71		– 0.01	0.89		– 0.03	0.53		0.03	0.50	
Sex	0.03	0.30		**– 0.12**	** < 0.001**		– 0.002	0.94		**– 0.22**	** < 0.001**	
Family SES	– 0.04	0.18		0.02	0.55		– 0.04	0.26		– 0.05	0.09	
Family structure	**0.07**	**0.03**		**0.07**	**0.02**		0.06	0.06		0.04	0.20	
Baseline maladjustment	**0.20**	** < 0.001**		**0.21**	** < 0.001**		**0.19**	** < 0.001**		**0.16**	** < 0.001**	

Baseline maladjustment reflects equivalent measure to outcome self-reported at age 11

*SR* self-report, *PR* parent-report, *TR* teacher-report, *PeerR* peer-report

Coefficients with *p* < .05 are bolded.

### Additional analyses

Descriptive statistics for variables not used in the main analyses are presented in Supplementary Table 1, together with information on age 13 assessments and attrition information.

### Severe peer victimization at age 11 and maladjustment at ages 13 through 29

*Supplementary Tables 2a-2f* depict adjusted associations between severe peer victimization at age 11 and maladjustment at ages 13 through 29. Parent-reported severe victimization was again the most consistent predictor of maladjustment symptoms but significant associations were fewer than in the main analyses and teacher-reported severe victimization predicted maladjustment symptoms later on nearly as often. For both, about half of the associations were still statistically significant in models containing all reporters. Self- and peer-reported severe peer victimization predicted maladjustment symptoms each in only 1 out of 24 models.

### Peer victimization at age 13 and maladjustment at ages 16 through 29

*Supplementary Tables 3a-3e* depict adjusted associations between peer victimization at age 13 and maladjustment symptoms at ages 16 through 29. Parent-reported victimization was a significant predictor in nearly all models, also when all reports were entered simultaneously. Teacher-reported victimization was a significant predictor in about half of all models, with better prediction of thought problems and somatic complaint. This is similar to self-reported victimization which relatively consistently predicted somatic complaints, though only in models where only one report was entered. As in main analyses, peer reports did not predict maladjustment symptoms.

### Maladjustment symptoms in chronically victimized adolescents

*Supplementary Tables 4a-4e* depict associations between chronic victimization, i.e., at least moderate victimization at both ages, and maladjustment. Again, parent-reported victimization was a stable predictor of maladjustment with associations remaining significant in models with all reports entered, except for withdrawal at age 22 and most age 29 outcomes. Self-reported chronic victimization was linked to most maladjustment symptoms in adolescence and early adulthood but associations were mostly not stable in combined models. Teacher-reported chronic victimization was not associated with later maladjustment, neither was peer-reported victimization.

### Peer victimization at age 11 and maladjustment symptoms at ages 13 through 29 conceptualized as latent variables

Finally, we computed all models with maladjustment symptoms conceptualized as latent variable (*Supplementary Table 5a-5f*). Models were otherwise identical to those including all reporters simultaneously, with the exception that baseline maladjustment was also conceptualized as latent factor. Parent-reported victimization significantly predicted the latent construct at all time points and all associations were retained in models including all reporters. Other results are similar to results for analyses in which facets of maladjustment were analysed separately in that teacher-reported victimization predicted some outcomes but self- and peer-reported victimization were no significant predictors of maladjustment.

## Discussion

We aimed to elucidate origins of heterogeneity in effect sizes reported for associations between peer victimization and maladjustment and focused on (1) interval between exposure and outcome and (2) reporter. Overall, peer victimization as reported by parents emerged as most stable predictor of maladjustment in adulthood, followed by teacher-reports which predicted maladjustment except for when victimization was conceptualized as occurring chronically. Self- and especially peer-reported victimization were hardly associated with adult maladjustment when baseline maladjustment, covariates, or other reporters were taken into account. Effect sizes for shorter versus longer intervals varied only marginally in our data.

The degree of overlap between reporters of peer victimization was modest like in other studies (e.g., [18]), which demonstrates that different reporters have different perspectives and that an individual who sees themselves as victim might not be perceived as such by others. Different to peers and, to a lesser degree teachers, parents are likely not present when the victimization happens, yet their accounts are most similar to adolescents’ own. The overlap between self- and parent-reported victimization suggests that adolescents do talk about their peer experiences at home, which is supported by studies on disclosing victimization [24]. Studies suggest that adolescents confide in their parents when the situation at school has become particularly bad [4, 21], which might explain why parents’ reports were most predictive of later maladjustment. That said, although parent-reported *severe* victimization also more often predicted maladjustment than other-reported severe victimization, effect sizes were more modest than in main analyses which could be an indication that parents do not just pick up the most severe victimization. Parental perception of their offspring may connect victimization and mental health: Parents who view their child as particularly vulnerable and sensitive might tend to suspect victimization or might interpret common conflict between young adolescents as teasing and bullying, and might also create a home environment for young people to be more likely to self-perceive mental health difficulties. These tentative interpretations must be tested in future research, to unravel how different sources of parental knowledge (e.g., child-initiated disclosure versus parental factors) differentially relate to maladjustment. Our results show that parents’ perceptions of victimization—sometimes dismissed as less reliable than reports by those present in the school context—should probably be given weight in research and practice.

Importantly, effect sizes were modest and the amount of variance explained remained firmly below 30% (except for some latent variable models) even though previous maladjustment symptoms and other widely studied precursors of maladjustment were included in analyses. Peer victimization might act indirectly on later mental health through physiological stress response, cognitive processes or substance use, but its direct effect on maladjustment appears to be less systematic than sometimes argued and hypothesized for the present study.

This is especially the case for associations between self-reported victimization and maladjustment symptoms. Tentatively, adolescents’ perceptions of being victimized might have been biased by existing maladjustment. Indeed, pairwise correlations between self-reported victimization and maladjustment were significant across all assessments but were substantially reduced when baseline maladjustment was added. Of course, experiences of peer victimization might well have started long before the age 11 assessment, especially as this age marks one of the final years of primary school in the Netherlands, and peer dynamics reported at that time likely reflect stable experiences. We cannot draw conclusions here as to whether peer victimization preceded maladjustment or vice versa; our interpretation can only go as far as noting that even if adolescents reported to be victimized at age 11, this did not worsen their maladjustment symptoms when maladjustment was present already. We note that self-reported chronic victimization predicted maladjustment at least in the short- to mid-term better than when victimization was conceptualized as snapshot, even when controlling for baseline. This suggests that victimization explains variance in worsening of maladjustment symptoms if it occurs (or is perceived to occur) over time and contexts.

Of note is also the absence of associations between peer-reported victimization and maladjustment symptoms for adjusted analyses and correlations where assessments were more than a couple of years apart. Peer nominations of victimizations seem trustworthy because they were associated with both concurrent and subsequent maladjustment. However, despite this form of assessment often being lauded as “gold standard” [13] for assessing victimization, peer reports might be more reflective of victims’ social status and as such be more of a reputational measure than one that taps into the psychological experience or later mental health risk for victims. Given that our findings actually mirrors empirical evidence [31], future research is needed to zoom in on potential correlates—or reasons for the absence—of peers’ perceptions of who is victimized. Also, replications in other data, ideally from different contexts or periods in time are needed to exclude the possibility that findings are specific to our data.

### Limitations

With respect to predictors, parent-reported victimization at age 11 referred to “teasing” rather than “bullying”. It is reassuring that concordance with other measures as associations with later maladjustment hardly differ from those involving victimization measured at 13 where the Child Behaviour Checklist referred to “bullying” directly. Related, the use of single items to assess bullying is not optimal, especially as no definition of bullying was given, the items captures neither specific forms nor duration or other elements deemed important definitional characteristics of bullying-victimization [11]. It is also possible that young people do not agree with the label bullying but would affirm the behaviours it entails. Thirdly, the teacher measure of peer victimization was not ideal as the vignette captures not only victimization but also awkward social behaviour. Nonetheless, these reports overlapped with self-, parent-, and peer-reported victimization in the expected range, thus can be considered a suitable proxy for assessing peer victimization from teachers.

With respect to outcomes, we used maladjustment symptoms as assessed in questionnaires but could not rely on clinical diagnoses, which would have increased the objectivity of assessments. Our selection of outcomes did not include other previously reported correlates of bullying-victimization such as suicidal ideation and suicide attempts [15] because this information was not repeatedly assessed in TRAILS. Having to rely on data availability is a limitation of secondary data analysis.

With respect to our sample, only about half of the sample took part in peer nominations. We also do not know whether some schools tackled bullying whereas others did not. We owe many of these limitations to the use of data collected at a time when bullying research was in its infancy and measurement by far not as developed as it is now. We certainly acknowledge the weaknesses of this measurement in terms of sensitivity and reliability but are grateful that researchers actually did assess bullying back then when most schools and policy makers hardly attended to this issue. Lastly, chronicity of victimization is predictive of its impact [30] but in our sample, groups of severely and chronically victimized adolescents were small. Larger sample sizes are needed to formally test chronicity and victimization across informants.

## Conclusion

Our data suggest that reporter *does* matter when studying peer victimization and its long-term correlates: parent-reported victimization is a moderate yet consistent predictor of maladjustment, whereas self- and peer-reported victimization were not stably associated with maladjustment when important covariates were considered. "Whom you ask" might deliver information that is relevant for different purposes but our results underline parents should be taken more seriously in research on bullying and victimization and in practical settings.

## Supplementary Information

Below is the link to the electronic supplementary material.

Supplementary file1 (DOCX 589 KB)

## Data Availability

Data are available through established TRAILS data request procedures. Analyses code is provide on the Open Science Framework page of the first author.
