# Supplementary material for: Peer victimization in early adolescence and maladjustment in adulthood
Source: Eur Child Adolesc Psychiatry. 2024 Jul 29;34(3):1011–24. doi: 10.1007/s00787-024-02532-5 (PMC11909069; doi:10.1007/s00787-024-02532-5)
Supplement: Supplementary file 1 — Supplementary file1 (DOCX 589 KB) [file 787_2024_2532_MOESM1_ESM.docx]

**Supplementary Table 1**

***Descriptive statistics for measures used in additional analyses***

|  | *n* | *Mean* | *SD* | *Range* |
| --- | --- | --- | --- | --- |
| **Predictors** | | | | |
| *Peer victimization at age 13* | | | | |
| Self-report^1^ | 2082 | 0.19 | 0.45 | 0.00 - 2.00 |
| Parent-report^2^ | 1918 | 0.16 | 0.39 | 0.00 - 2.00 |
| Teacher-report^3^ | 1537 | 0.68 | 1.02 | 0.00 - 4.00 |
| Peer-report | 1007 | 0.02 | 0.06 | 0.00 - 0.63 |
| *Severe peer victimization* | | | | |
|  | Severely victimized at age 11 | |  | |
| Self-report | 5.7% (*n* = 125) | |  | |
| Parent-report | 4.2% (*n* = 86) | |  | |
| Teacher-report | 6.1% (*n* = 118) | |  | |
| Peer-report | 10% (*n* = 110) | |  | |
| *Chronic peer victimization* | | | | |
| Self-report | 12.4% (*n* = 251) | |  | |
| Parent-report | 1.4% (*n* = 189) | |  | |
| Teacher-report | 12.8% (*n* = 149) | |  | |
| Peer-report | 7% (*n* = 51) | |  | |

**Note:** ^1^ Self-reported peer victimization at age 13 was assessed using one item from the Youth Self Report “I get bullied a lot”, rated from 0 = *not at all* to 2 = *often*. ^2^ Parent-reported peer victimization at age 13 was assessed using one item from the Child Behaviour Checklist “My child gets bullied a lot”, rated as 0 = *does not apply at all to* 2 = *applies often*. ^3^ Teacher-reported peer victimization at age 13 was assessed using a Social Problems vignette based on the Teachers Report Form (Achenbach, 1991) which included short vignettes including several traits and behaviours that correspond to the Youth Self Report and Child Behaviour Checklist. The Social Problems vignette contained descriptions that match peer victimization items such as “is teased a lot”. Teachers indicated how well the vignette described the child (0 = *does not apply at all* to 5 = *almost always applies)*. ^4^ Peer nominations of who is a victim of bullying were collected in classrooms with at least three TRAILS participants or two participants on the condition that both had also participated in T1 peer nominations. Peer victimization assessed at 13 was a predictor of participation at later assessments as follows: Participation at the age 19 assessment was more likely for those whose teachers (*t*(1509) = 2.86, *p* = .004) and peers (*t*(993) = 4.96, *p* < .001) had not indicated victimization, participation at the age 22 assessment was more likely for those whose teachers (*t*(1533) = 3.47, *p* < .001) and peers (*t*(1005) = 3.69, *p* < .001) had not indicated victimization, participation at the age 26 assessment was more likely for those whose teachers (*t*(1535) = 3.10, *p* = .002) and peers (*t*(1005) = 5.08, *p* < .001) had not indicated victimization, and, finally, participation at the age 29 assessment was more likely for those whose teachers (*t*(1535) = 2.70, *p* = .007) and peers (*t*(1005) = 3.49, *p* = .001) had not indicated victimization. None of the other victimization reports were associated with follow-up participation.

**Supplementary Table 2**

***2a: Associations between severe peer victimization at age 11 and maladjustment at age 13***

|  | | Withdrawal | | | | | | Anxiety | | | | | | Thought problems | | | | | | Somatic complaints | | |
| --- | --- | --- | --- | --- | --- | --- | --- | --- | --- | --- | --- | --- | --- | --- | --- | --- | --- | --- | --- | --- | --- | --- |
|  | | *ß* | | *p* | | *R^2^* | | *ß* | | *p* | | *R^2^* | | *ß* | | *p* | | *R^2^* | | *ß* | *p* | *R^2^* |
| **Separate models per reporter** | | | | | | | | | | | | | | | | | | | | | | |
| SR severe victimization | -.001 | | .99 | | .20 | | .02 | | .46 | | .26 | | -.03 | | .12 | | .18 | | .02 | | .37 | .23 |
| Sex | **-.14** | | **<.001** | |  | | **-.22** | | **<.001** | |  | | **-.12** | | **<.001** | |  | | **-.20** | | **<.001** |  |
| Family SES | -.003 | | .87 | |  | | .02 | | .34 | |  | | .02 | | .30 | |  | | **-.08** | | **<.001** |  |
| Family structure | **.04** | | **.03** | |  | | .02 | | .45 | |  | | **.07** | | **.001** | |  | | .01 | | .71 |  |
| Baseline maladjustment | **.42** | | **<.001** | |  | | **.44** | | **<.001** | |  | | **.40** | | **<.001** | |  | | **.40** | | **<.001** |  |
| PR severe victimization | **.08** | | **.001** | | .21 | | **.07** | | **.001** | | .26 | | .04 | | .07 | | .18 | | .04 | | .05 | .23 |
| Sex | **-.14** | | **<.001** | |  | | **-.22** | | **<.001** | |  | | **-.12** | | **<.001** | |  | | **-.20** | | **<.001** |  |
| Family SES | .01 | | .78 | |  | | .03 | | .20 | |  | | .03 | | .19 | |  | | **-.08** | | **<.001** |  |
| Family structure | **.05** | | **.03** | |  | | .02 | | .40 | |  | | **.07** | | **.001** | |  | | .01 | | .67 |  |
| Baseline maladjustment | **.41** | | **<.001** | |  | | **.43** | | **<.001** | |  | | **.39** | | **<.001** | |  | | **.40** | | **<.001** |  |
| TR severe victimization | **.06** | | **.01** | | .21 | | .01 | | .56 | | .26 | | .02 | | .26 | | .18 | | .03 | | .19 | .23 |
| Sex | **-.14** | | **<.001** | |  | | **-.22** | | **<.001** | |  | | **-.12** | | **<.001** | |  | | **-.20** | | **<.001** |  |
| Family SES | .004 | | .86 | |  | | .02 | | .32 | |  | | .03 | | .20 | |  | | **-.08** | | **<.001** |  |
| Family structure | **.04** | | **.04** | |  | | .02 | | .45 | |  | | **.07** | | **.001** | |  | | .01 | | .72 |  |
| Baseline maladjustment | **.41** | | **<.001** | |  | | **.44** | | **<.001** | |  | | **.39** | | **<.001** | |  | | **.40** | | **<.001** |  |
| PeerR severe victimization | .01 | | .68 | | .20 | | **.07** | | **.01** | | .27 | | .04 | | .21 | | .18 | | .05 | | .09 | .23 |
| Sex | **-.14** | | **<.001** | |  | | **-.22** | | **<.001** | |  | | **-.12** | | **<.001** | |  | | **-.20** | | **<.001** |  |
| Family SES | -.002 | | .91 | |  | | .02 | | .24 | |  | | .03 | | .21 | |  | | **-.08** | | **<.001** |  |
| Family structure | **.04** | | **.03** | |  | | .01 | | .53 | |  | | **.07** | | **.001** | |  | | .01 | | .76 |  |
| Baseline maladjustment | **.42** | | **<.001** | |  | | **.44** | | **<.001** | |  | | **.39** | | **<.001** | |  | | **.40** | | **<.001** |  |
| **Model with all reporters included simultaneously** | | | | | | | | | | | | | | | | | | | | | | |
| SR severe victimization | | -.03 | | .25 | | .21 | | -.01 | | .56 | | .27 | | -.05 | | .02 | | .18 | | <.001 | .98 | .23 |
| PR severe victimization | | **.07** | | **.001** | |  | | **.06** | | **.004** | |  | | .04 | | .08 | |  | | .03 | .21 |  |
| TR severe victimization | | .04 | | .08 | |  | | -.02 | | .45 | |  | | .01 | | .58 | |  | | .01 | .70 |  |
| PeerR severe victimization | | .01 | | .72 | |  | | **.08** | | **.01** | |  | | .04 | | .19 | |  | | .04 | .15 |  |
| Sex | | **-.14** | | **<.001** | |  | | **-.22** | | **<.001** | |  | | **-.12** | | **<.001** | |  | | **-.20** | **<.001** |  |
| Family SES | | .01 | | .64 | |  | | .03 | | .15 | |  | | .03 | | .15 | |  | | **-.08** | **<.001** |  |
| Family structure | | **.05** | | **.03** | |  | | .01 | | .49 | |  | | **.07** | | **.001** | |  | | .01 | .75 |  |
| Baseline maladjustment | | **.41** | | **<.001** | |  | | **.43** | | **<.001** | |  | | **.39** | | **<.001** | |  | | **.40** | **<.001** |  |

***Note***

SR = Self-report, PR=Parent-report, TR=Teacher-report, PeerR=Peer-report. Baseline maladjustment reflects equivalent measure to outcome self-reported at age 11. Coefficients with *p* < .05 are bolded.

***2b: Associations between severe peer victimization at age 11 and maladjustment at age 16***

|  | Withdrawal | | | Anxiety | | | Thought problems | | | Somatic complaints | | |  |
| --- | --- | --- | --- | --- | --- | --- | --- | --- | --- | --- | --- | --- | --- |
|  | *ß* | *p* | *R^2^* | *ß* | *p* | *R^2^* | *ß* | *p* | *R^2^* | *ß* | *p* | *R^2^* |  |
| **Separate models per reporter** | | | | | | | | | | | | | |
| SR severe victimization | .02 | .49 | .13 | .02 | .44 | .21 | .03 | .29 | .12 | .04 | .07 | .21 | |
| Sex | **-.12** | **<.001** |  | **-.28** | **<.001** |  | **-.08** | **<.001** |  | **-.31** | **<.001** |  | |
| Family SES | **-.06** | **.01** |  | -.01 | .59 |  | -.03 | .19 |  | **-.08** | **.001** |  | |
| Family structure | **.05** | **.03** |  | .03 | .23 |  | **.10** | **<.001** |  | **.05** | **.05** |  | |
| Baseline maladjustment | **.32** | **<.001** |  | **.33** | **<.001** |  | **.31** | **<.001** |  | **.28** | **<.001** |  | |
| PR severe victimization | **.07** | **.01** | .13 | **.05** | **.02** | .22 | .05 | .05 | .12 | **.06** | **.02** | .21 | |
| Sex | **-.11** | **<.001** |  | **-.28** | **<.001** |  | **-.08** | **.001** |  | **-.31** | **<.001** |  | |
| Family SES | **-.05** | **.03** |  | -.01 | .75 |  | -.03 | .26 |  | **-.07** | **.001** |  | |
| Family structure | **.06** | **.02** |  | .03 | .21 |  | **.10** | **<.001** |  | .05 | .05 |  | |
| Baseline maladjustment | **.31** | **<.001** |  | **.33** | **<.001** |  | **.31** | **<.001** |  | **.28** | **<.001** |  | |
| TR severe victimization | .03 | .19 | .13 | .04 | .07 | .21 | **.06** | **.03** | .12 | .05 | .05 | .21 | |
| Sex | **-.12** | **<.001** |  | **-.28** | **<.001** |  | **-.08** | **<.001** |  | **-.31** | **<.001** |  | |
| Family SES | **-.06** | **.02** |  | -.01 | .70 |  | -.03 | .26 |  | **-.08** | **.001** |  | |
| Family structure | **.05** | **.03** |  | .03 | .26 |  | **.10** | **<.001** |  | .04 | .06 |  | |
| Baseline maladjustment | **.32** | **<.001** |  | **.33** | **<.001** |  | **.31** | **<.001** |  | **.28** | **<.001** |  | |
| PeerR severe victimization | -.004 | .91 | .13 | -.01 | .70 | .21 | -.04 | .18 | .12 | .01 | .70 | .20 | |
| Sex | **-.11** | **<.001** |  | **-.28** | **<.001** |  | **-.08** | **.001** |  | **-.31** | **<.001** |  | |
| Family SES | **-.06** | **.01** |  | -.01 | .54 |  | -.04 | .14 |  | **-.08** | **.001** |  | |
| Family structure | **.05** | **.03** |  | .03 | .22 |  | **.10** | **<.001** |  | .05 | .05 |  | |
| Baseline maladjustment | **.32** | **<.001** |  | **.34** | **<.001** |  | **.31** | **<.001** |  | **.28** | **<.001** |  | |
| **Model with all reporters included simultaneously** | | | | | | | | | | | | | |
| SR severe victimization | -.001 | .96 | .14 | .002 | .93 | .22 | .02 | .56 | .13 | .02 | .34 | .21 |  |
| PR severe victimization | **.07** | **.01** |  | **.05** | **.04** |  | .04 | .10 |  | .05 | .08 |  |  |
| TR severe victimization | .02 | .53 |  | .03 | .28 |  | .05 | .05 |  | .03 | .24 |  |  |
| PeerR severe victimization | -.01 | .84 |  | -.004 | .89 |  | -.05 | .13 |  | .002 | .96 |  |  |
| Sex | **-.12** | **<.001** |  | **-.28** | **<.001** |  | **-.08** | **<.001** |  | **-.31** | **<.001** |  |  |
| Family SES | **-.05** | **.03** |  | -.01 | .82 |  | -.03 | .29 |  | **-.07** | **.002** |  |  |
| Family structure | **.05** | **.03** |  | .03 | .23 |  | **.10** | **<.001** |  | .04 | .06 |  |  |
| Baseline maladjustment | **.31** | **<.001** |  | **.33** | **<.001** |  | **.31** | **<.001** |  | **.28** | **<.001** |  |  |

***Note***

SR = Self-report, PR=Parent-report, TR=Teacher-report, PeerR=Peer-report. Baseline maladjustment reflects equivalent measure to outcome self-reported at age 11. Coefficients with *p* < .05 are bolded.

***2c: Associations between severe peer victimization at age 11 and maladjustment at age 19***

|  | | Withdrawal | | | | | | Anxiety | | | | | | Thought problems | | | | | Somatic complaints | | |  |
| --- | --- | --- | --- | --- | --- | --- | --- | --- | --- | --- | --- | --- | --- | --- | --- | --- | --- | --- | --- | --- | --- | --- |
|  | | *ß* | | *p* | | *R^2^* | | *ß* | | *p* | | *R^2^* | | *ß* | | *p* | | *R^2^* | *ß* | *p* | *R^2^* |  |
| **Separate models per reporter** | | | | | | | | | | | | | | | | | | | | | | |
| SR severe victimization | .03 | | .32 | | .09 | | .03 | | .19 | | .15 | | .05 | | .05 | | .09 | | .05 | .06 | .11 | |
| Sex | -.001 | | .95 | |  | | **-.15** | | **<.001** | |  | | -.02 | | .48 | |  | | **-.22** | **<.001** |  | |
| Family SES | **-.05** | | **.04** | |  | | -.02 | | .52 | |  | | -.03 | | .19 | |  | | **-.08** | **.001** |  | |
| Family structure | **.10** | | **<.001** | |  | | **.10** | | **<.001** | |  | | **.12** | | **<.001** | |  | | **.07** | **.003** |  | |
| Baseline maladjustment | **.25** | | **<.001** | |  | | **.31** | | **<.001** | |  | | **.25** | | **<.001** | |  | | **.18** | **<.001** |  | |
| PR severe victimization | **.06** | | **.03** | | .09 | | **.06** | | **.02** | | .15 | | .05 | | .07 | | .09 | | **.06** | **.02** | .11 | |
| Sex | -.002 | | .95 | |  | | **-.15** | | **<.001** | |  | | -.02 | | .50 | |  | | **-.22** | **<.001** |  | |
| Family SES | -.05 | | .07 | |  | | -.01 | | .68 | |  | | -.03 | | .23 | |  | | **-.08** | **.002** |  | |
| Family structure | **.10** | | **<.001** | |  | | **.10** | | **<.001** | |  | | **.12** | | **<.001** | |  | | **.07** | **.003** |  | |
| Baseline maladjustment | **.25** | | **<.001** | |  | | **.31** | | **<.001** | |  | | **.25** | | **<.001** | |  | | **.19** | **<.001** |  | |
| TR severe victimization | **.09** | | **<.001** | | .10 | | **.09** | | **.001** | | .15 | | **.12** | | **<.001** | | .10 | | **.13** | **<.001** | .12 | |
| Sex | -.01 | | .79 | |  | | **-.16** | | **<.001** | |  | | -.02 | | .33 | |  | | **-.23** | **<.001** |  | |
| Family SES | -.04 | | .09 | |  | | -.01 | | .76 | |  | | -.02 | | .39 | |  | | **-.07** | **.01** |  | |
| Family structure | **.09** | | **<.001** | |  | | **.10** | | **<.001** | |  | | **.12** | | **<.001** | |  | | **.07** | **.01** |  | |
| Baseline maladjustment | **.25** | | **<.001** | |  | | **.31** | | **<.001** | |  | | **.25** | | **<.001** | |  | | **.18** | **<.001** |  | |
| PeerR severe victimization | .02 | | .66 | | .09 | | -.02 | | .63 | | .15 | | -.001 | | .98 | | .09 | | -.02 | .65 | .11 | |
| Sex | -.001 | | .96 | |  | | **-.15** | | **<.001** | |  | | -.02 | | .51 | |  | | **-.22** | **<.001** |  | |
| Family SES | -.05 | | .05 | |  | | -.02 | | .45 | |  | | -.04 | | .17 | |  | | **-.08** | **.001** |  | |
| Family structure | **.09** | | **<.001** | |  | | **.10** | | **<.001** | |  | | **.12** | | **<.001** | |  | | **.07** | **.003** |  | |
| Baseline maladjustment | **.26** | | **<.001** | |  | | **.32** | | **<.001** | |  | | **.26** | | **<.001** | |  | | **.19** | **<.001** |  | |
| **Model with all reporters included simultaneously** | | | | | | | | | | | | | | | | | | | | | | |
| SR severe victimization | | -.002 | | .93 | | .10 | | .01 | | .60 | | .16 | | .03 | | .28 | | .10 | .02 | .40 | .13 |  |
| PR severe victimization | | .04 | | .12 | |  | | .05 | | .07 | |  | | .02 | | .54 | |  | .03 | .33 |  |  |
| TR severe victimization | | **.08** | | **.01** | |  | | **.08** | | **.01** | |  | | **.12** | | **<.001** | |  | **.13** | **<.001** |  |  |
| PeerR severe victimization | | .01 | | .75 | |  | | -.03 | | .47 | |  | | -.02 | | .65 | |  | -.04 | .32 |  |  |
| Sex | | -.01 | | .79 | |  | | **-.16** | | **<.001** | |  | | -.02 | | .33 | |  | -.23 | **<.001** |  |  |
| Family SES | | -.04 | | .13 | |  | | -.01 | | .83 | |  | | -.02 | | .42 | |  | -.07 | **.01** |  |  |
| Family structure | | **.09** | | **<.001** | |  | | **.10** | | **<.001** | |  | | **.12** | | **<.001** | |  | **.07** | **.01** |  |  |
| Baseline maladjustment | | **.25** | | **<.001** | |  | | **.30** | | **<.001** | |  | | **.24** | | **<.001** | |  | **.18** | **<.001** |  |  |

***Note***

SR = Self-report, PR=Parent-report, TR=Teacher-report, PeerR=Peer-report. Baseline maladjustment reflects equivalent measure to outcome self-reported at age 11. Coefficients with *p* < .05 are bolded.

***2d: Associations between severe peer victimization at age 11 and maladjustment at age 22***

|  | | Withdrawal | | | | | | Anxiety | | | | | | Thought problems | | | | | Somatic complaints | | |  |
| --- | --- | --- | --- | --- | --- | --- | --- | --- | --- | --- | --- | --- | --- | --- | --- | --- | --- | --- | --- | --- | --- | --- |
|  | | *ß* | | *p* | | *R^2^* | | *ß* | | *p* | | *R^2^* | | *ß* | | *p* | | *R^2^* | *ß* | *p* | *R^2^* |  |
| **Separate models per reporter** | | | | | | | | | | | | | | | | | | | | | | |
| SR severe victimization | .01 | | .64 | | .06 | | -.003 | | .91 | | .10 | | -.003 | | .92 | | .06 | | .02 | .37 | .14 | |
| Sex | .01 | | .75 | |  | | **-.15** | | **<.001** | |  | | -.03 | | .22 | |  | | **-.29** | **<.001** |  | |
| Family SES | -.05 | | .07 | |  | | .01 | | .71 | |  | | **-.06** | | **.02** | |  | | **-.07** | **.004** |  | |
| Family structure | .05 | | .06 | |  | | **.06** | | **.02** | |  | | **.08** | | **.003** | |  | | .05 | .07 |  | |
| Baseline maladjustment | **.23** | | **<.001** | |  | | **.25** | | **<.001** | |  | | **.21** | | **<.001** | |  | | **.19** | **<.001** |  | |
| PR severe victimization | .05 | | .09 | | .06 | | .05 | | .09 | | .10 | | **.06** | | **.04** | | .06 | | .05 | .06 | .14 | |
| Sex | .01 | | .75 | |  | | **-.16** | | **<.001** | |  | | -.03 | | .20 | |  | | **-.29** | **<.001** |  | |
| Family SES | -.04 | | .11 | |  | | .02 | | .56 | |  | | **-.06** | | **.04** | |  | | **-.07** | **.01** |  | |
| Family structure | .05 | | .05 | |  | | **.06** | | **.01** | |  | | **.08** | | **.003** | |  | | .05 | .07 |  | |
| Baseline maladjustment | **.22** | | **<.001** | |  | | **.24** | | **<.001** | |  | | **.21** | | **<.001** | |  | | **.19** | **<.001** |  | |
| TR severe victimization | .03 | | .39 | | .06 | | -.001 | | .96 | | .10 | | .002 | | .94 | | .06 | | .05 | .06 | .15 | |
| Sex | .01 | | .78 | |  | | **-.15** | | **<.001** | |  | | -.03 | | .21 | |  | | **-.29** | **<.001** |  | |
| Family SES | -.05 | | .09 | |  | | .01 | | .71 | |  | | **-.06** | | **.03** | |  | | **-.07** | **.01** |  | |
| Family structure | .05 | | .06 | |  | | **.06** | | **.02** | |  | | **.08** | | **.003** | |  | | .05 | .08 |  | |
| Baseline maladjustment | **.23** | | **<.001** | |  | | **.25** | | **<.001** | |  | | **.21** | | **<.001** | |  | | **.19** | **<.001** |  | |
| PeerR severe victimization | <.001 | | .99 | | .06 | | -.002 | | .96 | | .10 | | .05 | | .19 | | .06 | | -.02 | .55 | .14 | |
| Sex | .01 | | .74 | |  | | **-.15** | | **<.001** | |  | | -.03 | | .19 | |  | | **-.29** | **<.001** |  | |
| Family SES | -.05 | | .07 | |  | | .01 | | .70 | |  | | **-.06** | | **.03** | |  | | **-.08** | **.003** |  | |
| Family structure | .05 | | .06 | |  | | **.06** | | **.02** | |  | | **.08** | | **.003** | |  | | .05 | .07 |  | |
| Baseline maladjustment | **.23** | | **<.001** | |  | | **.25** | | **<.001** | |  | | **.20** | | **<.001** | |  | | **.20** | **<.001** |  | |
| **Model with all reporters included simultaneously** | | | | | | | | | | | | | | | | | | | | | | |
| SR severe victimization | | -.001 | | .96 | | .06 | | -.01 | | .64 | | .10 | | -.02 | | .39 | | .07 | .01 | .65 | .15 |  |
| PR severe victimization | | .05 | | .13 | |  | | .05 | | .07 | |  | | **.06** | | **.04** | |  | .04 | .16 |  |  |
| TR severe victimization | | .01 | | .67 | |  | | -.02 | | .63 | |  | | -.02 | | .55 | |  | .05 | .14 |  |  |
| PeerR severe victimization | | .01 | | .89 | |  | | .01 | | .87 | |  | | .05 | | .20 | |  | -.04 | .32 |  |  |
| Sex | | .01 | | .78 | |  | | **-.15** | | **<.001** | |  | | -.03 | | .21 | |  | **-.29** | **<.001** |  |  |
| Family SES | | -.04 | | .13 | |  | | .01 | | .61 | |  | | -.05 | | .05 | |  | **-.07** | **.01** |  |  |
| Family structure | | .05 | | .05 | |  | | **.06** | | **.01** | |  | | **.08** | | **.003** | |  | .05 | .07 |  |  |
| Baseline maladjustment | | **.22** | | **<.001** | |  | | **.24** | | **<.001** | |  | | **.21** | | **<.001** | |  | **.19** | **<.001** |  |  |

***Note***

SR = Self-report, PR=Parent-report, TR=Teacher-report, PeerR=Peer-report. Baseline maladjustment reflects equivalent measure to outcome self-reported at age 11. Coefficients with *p* < .05 are bolded.

***2e: Associations between severe peer victimization at age 11 and maladjustment at age 26***

|  | | Withdrawal | | | | | | Anxiety | | | | | | Thought problems | | | | | Somatic complaints | | |  |
| --- | --- | --- | --- | --- | --- | --- | --- | --- | --- | --- | --- | --- | --- | --- | --- | --- | --- | --- | --- | --- | --- | --- |
|  | | *ß* | | *p* | | *R^2^* | | *ß* | | *p* | | *R^2^* | | *ß* | | *p* | | *R^2^* | *ß* | *p* | *R^2^* |  |
| **Separate models per reporter** | | | | | | | | | | | | | | | | | | | | | | |
| SR severe victimization | .04 | | .22 | | .08 | | .01 | | .74 | | .09 | | .01 | | .82 | | .07 | | .03 | .33 | .10 | |
| Sex | .05 | | .05 | |  | | **-.11** | | **<.001** | |  | | .02 | | .46 | |  | | **-.25** | **<.001** |  | |
| Family SES | **-.06** | | **.03** | |  | | .03 | | .28 | |  | | -.03 | | .29 | |  | | **-.08** | **.01** |  | |
| Family structure | **.10** | | **<.001** | |  | | **.08** | | **.01** | |  | | **.12** | | **<.001** | |  | | .03 | .26 |  | |
| Baseline maladjustment | **.23** | | **<.001** | |  | | **.25** | | **<.001** | |  | | **.21** | | **<.001** | |  | | **.15** | **<.001** |  | |
| PR severe victimization | **.07** | | **.01** | | .09 | | .03 | | .35 | | .09 | | .05 | | .12 | | .07 | | .06 | .05 | .11 | |
| Sex | .05 | | .05 | |  | | **-.11** | | **<.001** | |  | | .02 | | .47 | |  | | **-.25** | **<.001** |  | |
| Family SES | **-.06** | | **.04** | |  | | .03 | | .25 | |  | | -.03 | | .38 | |  | | **-.07** | **.01** |  | |
| Family structure | **.10** | | **<.001** | |  | | **.08** | | **.01** | |  | | **.12** | | **<.001** | |  | | .03 | .27 |  | |
| Baseline maladjustment | **.22** | | **<.001** | |  | | **.25** | | **<.001** | |  | | **.20** | | **<.001** | |  | | **.16** | **<.001** |  | |
| TR severe victimization | **.07** | | **.03** | | .08 | | .05 | | .14 | | .09 | | **.08** | | **.02** | | .07 | | **.10** | **.001** | .11 | |
| Sex | .05 | | .07 | |  | | **-.11** | | **<.001** | |  | | .02 | | .58 | |  | | **-.26** | **<.001** |  | |
| Family SES | -.06 | | .05 | |  | | .04 | | .20 | |  | | -.02 | | .49 | |  | | **-.07** | **.02** |  | |
| Family structure | **.10** | | **.001** | |  | | **.07** | | **.01** | |  | | **.12** | | **<.001** | |  | | .02 | .40 |  | |
| Baseline maladjustment | **.23** | | **<.001** | |  | | **.25** | | **<.001** | |  | | **.20** | | **<.001** | |  | | **.16** | **<.001** |  | |
| PeerR severe victimization | .02 | | .67 | | .08 | | .01 | | .80 | | .09 | | .01 | | .80 | | .07 | | .05 | .20 | .11 | |
| Sex | .05 | | .05 | |  | | **-.11** | | **<.001** | |  | | .02 | | .47 | |  | | **-.25** | **<.001** |  | |
| Family SES | **-.06** | | **.02** | |  | | .03 | | .28 | |  | | -.03 | | .30 | |  | | **-.08** | **.01** |  | |
| Family structure | **.10** | | **<.001** | |  | | **.08** | | **.01** | |  | | **.12** | | **<.001** | |  | | .03 | .29 |  | |
| Baseline maladjustment | **.23** | | **<.001** | |  | | **.25** | | **<.001** | |  | | **.21** | | **<.001** | |  | | **.15** | **<.001** |  | |
| **Model with all reporters included simultaneously** | | | | | | | | | | | | | | | | | | | | | | |
| SR severe victimization | | .01 | | .79 | | .09 | | -.004 | | .91 | | .09 | | -.02 | | .61 | | .07 | -.01 | .82 | .12 |  |
| PR severe victimization | | .06 | | .05 | |  | | .02 | | .63 | |  | | .03 | | .34 | |  | .02 | .45 |  |  |
| TR severe victimization | | .05 | | .19 | |  | | .04 | | .21 | |  | | .07 | | .05 | |  | **.08** | **.01** |  |  |
| PeerR severe victimization | | .003 | | .94 | |  | | .01 | | .89 | |  | | .001 | | .98 | |  | .05 | .25 |  |  |
| Sex | | .05 | | .07 | |  | | **-.11** | | **<.001** | |  | | .02 | | .58 | |  | **-.26** | **<.001** |  |  |
| Family SES | | -.05 | | .07 | |  | | .04 | | .19 | |  | | -.02 | | .52 | |  | **-.06** | **.03** |  |  |
| Family structure | | **.10** | | **<.001** | |  | | **.07** | | **.01** | |  | | **.12** | | **<.001** | |  | .02 | .40 |  |  |
| Baseline maladjustment | | **.22** | | **<.001** | |  | | **.24** | | **<.001** | |  | | **.20** | | **<.001** | |  | **.15** | **<.001** |  |  |

***Note***

SR = Self-report, PR=Parent-report, TR=Teacher-report, PeerR=Peer-report. Baseline maladjustment reflects equivalent measure to outcome self-reported at age 11. Coefficients with *p* < .05 are bolded.

***2f: Associations between severe peer victimization at age 11 and maladjustment at age 29***

|  | | Withdrawal | | | | | | Anxiety | | | | | | Thought problems | | | | | Somatic complaints | | |  |
| --- | --- | --- | --- | --- | --- | --- | --- | --- | --- | --- | --- | --- | --- | --- | --- | --- | --- | --- | --- | --- | --- | --- |
|  | | *ß* | | *p* | | *R^2^* | | *ß* | | *p* | | *R^2^* | | *ß* | | *p* | | *R^2^* | *ß* | *p* | *R^2^* |  |
| **Separate models per reporter** | | | | | | | | | | | | | | | | | | | | | | |
| SR severe victimization | .02 | | .47 | | .07 | | -.01 | | .79 | | .08 | | -.02 | | .59 | | .06 | | .001 | .97 | .09 | |
| Sex | .05 | | .12 | |  | | **-.10** | | **.001** | |  | | .01 | | .75 | |  | | **-.21** | **<.001** |  | |
| Family SES | **-.07** | | **.03** | |  | | -.001 | | .98 | |  | | -.06 | | .08 | |  | | **-.08** | **.01** |  | |
| Family structure | **.08** | | **.01** | |  | | **.08** | | **.01** | |  | | **.07** | | **.02** | |  | | .05 | .08 |  | |
| Baseline maladjustment | **.22** | | **<.001** | |  | | **.23** | | **<.001** | |  | | **.21** | | **<.001** | |  | | **.16** | **<.001** |  | |
| PR severe victimization | **.08** | | **.02** | | .08 | | .05 | | .13 | | .08 | | .03 | | .41 | | .06 | | **.08** | **.01** | .10 | |
| Sex | .05 | | .13 | |  | | **-.11** | | **<.001** | |  | | .01 | | .80 | |  | | **-.21** | **<.001** |  | |
| Family SES | -.06 | | .07 | |  | | .01 | | .82 | |  | | -.05 | | .12 | |  | | **-.06** | **.04** |  | |
| Family structure | **.08** | | **.01** | |  | | **.08** | | **.01** | |  | | **.07** | | **.02** | |  | | .05 | .09 |  | |
| Baseline maladjustment | **.22** | | **<.001** | |  | | **.22** | | **<.001** | |  | | **.20** | | **<.001** | |  | | **.16** | **<.001** |  | |
| TR severe victimization | .07 | | .06 | | .08 | | .06 | | .13 | | .08 | | .05 | | .20 | | .06 | | **.08** | **.03** | .09 | |
| Sex | .04 | | .15 | |  | | **-.11** | | **<.001** | |  | | .01 | | .85 | |  | | **-.21** | **<.001** |  | |
| Family SES | -.06 | | .05 | |  | | .01 | | .86 | |  | | -.05 | | .12 | |  | | **-.07** | **.03** |  | |
| Family structure | **.08** | | **.01** | |  | | **.08** | | **.01** | |  | | **.07** | | **.03** | |  | | .05 | .12 |  | |
| Baseline maladjustment | **.22** | | **<.001** | |  | | **.23** | | **<.001** | |  | | **.20** | | **<.001** | |  | | **.16** | **<.001** |  | |
| PeerR severe victimization | .07 | | .10 | | .08 | | .02 | | .57 | | .08 | | -.01 | | .76 | | .05 | | .06 | .17 | .09 | |
| Sex | .04 | | .15 | |  | | **-.10** | | **<.001** | |  | | .01 | | .76 | |  | | **-.21** | **<.001** |  | |
| Family SES | -.06 | | .05 | |  | | .002 | | .95 | |  | | -.05 | | .09 | |  | | **-.07** | **.02** |  | |
| Family structure | **.08** | | **.01** | |  | | **.08** | | **.01** | |  | | **.07** | | **.02** | |  | | .05 | .09 |  | |
| Baseline maladjustment | **.22** | | **<.001** | |  | | **.23** | | **<.001** | |  | | **.21** | | **<.001** | |  | | **.16** | **<.001** |  | |
| **Model with all reporters included simultaneously** | | | | | | | | | | | | | | | | | | | | | | |
| SR severe victimization | | -.02 | | .60 | | .08 | | -.03 | | .33 | | .08 | | -.03 | | .32 | | .06 | -.04 | .24 | .10 |  |
| PR severe victimization | | **.08** | | **.04** | |  | | .05 | | .17 | |  | | .04 | | .33 | |  | **.07** | **.04** |  |  |
| TR severe peer victimization | | .04 | | .37 | |  | | .04 | | .33 | |  | | .04 | | .28 | |  | .05 | .24 |  |  |
| PeerR severe victimization | | .06 | | .16 | |  | | .02 | | .67 | |  | | -.01 | | .80 | |  | .04 | .31 |  |  |
| Sex | | .04 | | .18 | |  | | **-.11** | | **<.001** | |  | | .01 | | .81 | |  | **-.21** | **<.001** |  |  |
| Family SES | | -.05 | | .10 | |  | | .01 | | .76 | |  | | -.05 | | .13 | |  | -.06 | .05 |  |  |
| Family structure | | **.08** | | **.01** | |  | | **.08** | | **.01** | |  | | **.07** | | **.03** | |  | .05 | .12 |  |  |
| Baseline maladjustment | | **.21** | | **<.001** | |  | | **.23** | | **<.001** | |  | | **.20** | | **<.001** | |  | **.16** | **<.001** |  |  |

***Note***

SR = Self-report, PR=Parent-report, TR=Teacher-report, PeerR=Peer-report. Baseline maladjustment reflects equivalent measure to outcome self-reported at age 11. Coefficients with *p* < .05 are bolded.

**Supplementary Table 3**

***3a: Associations between peer victimization at age 13 and maladjustment at age 16***

|  | Withdrawal | | | Anxiety | | | Thought problems | | | Somatic complaints | | |
| --- | --- | --- | --- | --- | --- | --- | --- | --- | --- | --- | --- | --- |
|  | *ß* | *p* | *R^2^* | *ß* | *p* | *R^2^* | *ß* | *p* | *R^2^* | *ß* | *p* | *R^2^* |
| **Separate models per reporter** | | | | | | | | | | | | |
| SR peer victimization | .04 | .11 | .28 | -.01 | .77 | .34 | **.06** | **.01** | .21 | **.04** | **.04** | .30 |
| Sex | **-.06** | **.01** |  | **-.19** | **<.001** |  | -.04 | .10 |  | **-.24** | **<.001** |  |
| Family SES | **-.05** | **.02** |  | -.02 | .37 |  | -.03 | .16 |  | -.04 | .10 |  |
| Family structure | .04 | .07 |  | .03 | .15 |  | **.08** | **<.001** |  | **.04** | **.04** |  |
| Baseline maladjustment | **.50** | **<.001** |  | **.50** | **<.001** |  | **.42** | **<.001** |  | **.43** | **<.001** |  |
| PR peer victimization | **.05** | **.04** | .28 | **.08** | **<.001** | .34 | **.07** | **.00** | .21 | **.10** | **<.001** | .31 |
| Sex | **-.06** | **.01** |  | **-.20** | **<.001** |  | -.04 | .08 |  | **-.24** | **<.001** |  |
| Family SES | **-.05** | **.02** |  | -.01 | .68 |  | -.03 | .17 |  | -.03 | .15 |  |
| Family structure | .04 | .09 |  | .03 | .20 |  | **.08** | **.001** |  | .04 | .07 |  |
| Baseline maladjustment | **.49** | **<.001** |  | **.48** | **<.001** |  | **.42** | **<.001** |  | **.43** | **<.001** |  |
| TR peer victimization | .01 | .75 | .28 | -.01 | .56 | .34 | .02 | .51 | .21 | **.06** | **.01** | .31 |
| Sex | **-.06** | **.01** |  | **-.19** | **<.001** |  | -.04 | .11 |  | **-.24** | **<.001** |  |
| Family SES | **-.06** | **.01** |  | -.02 | .37 |  | -.04 | .10 |  | -.04 | .10 |  |
| Family structure | .04 | .08 |  | .03 | .15 |  | **.08** | **<.001** |  | .04 | .06 |  |
| Baseline maladjustment | **.50** | **<.001** |  | **.50** | **<.001** |  | **.43** | **<.001** |  | **.43** | **<.001** |  |
| PeerR peer victimization | .01 | .75 | .28 | .06 | .08 | .34 | .03 | .35 | .21 | .06 | .07 | .31 |
| Sex | **-.06** | **.01** |  | **-.20** | **<.001** |  | -.04 | .09 |  | **-.24** | **<.001** |  |
| Family SES | **-.06** | **.01** |  | -.01 | .66 |  | -.04 | .14 |  | -.03 | .15 |  |
| Family structure | .04 | .09 |  | .03 | .22 |  | **.08** | **.001** |  | .04 | .08 |  |
| Baseline maladjustment | **.51** | **<.001** |  | **.50** | **<.001** |  | **.43** | **<.001** |  | **.43** | **<.001** |  |
| **Model with all reporters included simultaneously** | | | | | | | | | | | | |
| SR peer victimization | .02 | .35 | .28 | -.04 | .08 | .35 | .04 | .18 | .21 | .003 | .92 | .31 |
| PR peer victimization | .04 | .11 |  | **.10** | **<.001** |  | **.06** | **.03** |  | **.07** | **.01** |  |
| TR peer victimization | -.02 | .54 |  | -.05 | .06 |  | -.01 | .72 |  | .03 | .33 |  |
| PeerR peer victimization | .003 | .94 |  | .05 | .11 |  | .01 | .85 |  | .03 | .46 |  |
| Sex | **-.06** | **.01** |  | **-.20** | **<.001** |  | -.04 | .09 |  | **-.25** | **<.001** |  |
| Family SES | **-.05** | **.03** |  | -.01 | .74 |  | -.03 | .24 |  | -.03 | .23 |  |
| Family structure | .04 | .08 |  | .02 | .27 |  | **.08** | **.001** |  | .04 | .08 |  |
| Baseline maladjustment | **.49** | **<.001** |  | **.50** | **<.001** |  | **.42** | **<.001** |  | **.42** | **<.001** |  |

***Note***

SR = Self-report, PR=Parent-report, TR=Teacher-report, PeerR=Peer-report. Baseline maladjustment reflects equivalent measure to outcome self-reported at age 11. Coefficients with *p* < .05 are bolded.

***3b: Associations between peer victimization at age 13 and maladjustment at age 19***

|  | Withdrawal | | | Anxiety | | | Thought problems | | | Somatic complaints | | |
| --- | --- | --- | --- | --- | --- | --- | --- | --- | --- | --- | --- | --- |
|  | *ß* | *p* | *R^2^* | *ß* | *p* | *R^2^* | *ß* | *p* | *R^2^* | *ß* | *p* | *R^2^* |
| **Separate models per reporter** | | | | | | | | | | | | |
| SR peer victimization | .002 | .93 | .16 | .01 | .76 | .21 | **.05** | **.03** | .13 | .02 | .44 | .13 |
| Sex | .05 | .05 |  | **-.08** | **<.001** |  | .02 | .40 |  | **-.18** | **<.001** |  |
| Family SES | **-.05** | **.03** |  | -.02 | .42 |  | -.03 | .17 |  | **-.06** | **.02** |  |
| Family structure | **.08** | **<.001** |  | **.09** | **<.001** |  | **.11** | **<.001** |  | **.07** | **.004** |  |
| Baseline maladjustment | **.38** | **<.001** |  | **.42** | **<.001** |  | **.32** | **<.001** |  | **.25** | **<.001** |  |
| PR peer victimization | **.08** | **.001** | .17 | **.14** | **<.001** | .23 | **.17** | **<.001** | .15 | **.12** | **<.001** | .15 |
| Sex | .04 | .09 |  | **-.10** | **<.001** |  | .01 | .63 |  | **-.19** | **<.001** |  |
| Family SES | -.04 | .08 |  | -.002 | .92 |  | -.02 | .39 |  | **-.05** | **.04** |  |
| Family structure | **.08** | **.001** |  | **.09** | **<.001** |  | **.10** | **<.001** |  | **.06** | **.01** |  |
| Baseline maladjustment | **.36** | **<.001** |  | **.39** | **<.001** |  | **.31** | **<.001** |  | **.24** | **<.001** |  |
| TR peer victimization | .03 | .29 | .16 | .04 | .14 | .22 | **.12** | **<.001** | .14 | **.08** | **.003** | .14 |
| Sex | .04 | .07 |  | **-.09** | **<.001** |  | .01 | .70 |  | **-.19** | **<.001** |  |
| Family SES | **-.05** | **.04** |  | -.02 | .46 |  | -.03 | .17 |  | **-.06** | **.02** |  |
| Family structure | **.08** | **.001** |  | **.09** | **<.001** |  | **.10** | **<.001** |  | **.06** | **.01** |  |
| Baseline maladjustment | **.38** | **<.001** |  | **.41** | **<.001** |  | **.31** | **<.001** |  | **.25** | **<.001** |  |
| PeerR peer victimization | -.04 | .29 | .16 | -.02 | .66 | .21 | .08 | .06 | .13 | .01 | .78 | .13 |
| Sex | **.05** | **.03** |  | **-.08** | **<.001** |  | .01 | .55 |  | **-.18** | **<.001** |  |
| Family SES | **-.06** | **.02** |  | -.02 | .37 |  | -.03 | .23 |  | **-.06** | **.02** |  |
| Family structure | **.09** | **<.001** |  | **.10** | **<.001** |  | **.10** | **<.001** |  | **.07** | **.01** |  |
| Baseline maladjustment | **.39** | **<.001** |  | **.42** | **<.001** |  | **.33** | **<.001** |  | **.25** | **<.001** |  |
| **Model with all reporters included simultaneously** | | | | | | | | | | | | |
| SR peer victimization | -.02 | .37 | .17 | -.04 | .12 | .23 | -.03 | .20 | .16 | -.03 | .23 | .15 |
| PR peer victimization | **.09** | **.002** |  | **.15** | **<.001** |  | **.14** | **<.001** |  | **.10** | **<.001** |  |
| TR peer victimization | .02 | .56 |  | .01 | .87 |  | **.07** | **.02** |  | **.07** | **.03** |  |
| PeerR peer victimization | -.04 | .30 |  | -.04 | .29 |  | .05 | .20 |  | -.02 | .61 |  |
| Sex | .04 | .06 |  | **-.09** | **<.001** |  | <.001 | .98 |  | **-.19** | **<.001** |  |
| Family SES | **-.05** | **.04** |  | -.01 | .62 |  | -.02 | .53 |  | **-.05** | **.03** |  |
| Family structure | **.08** | **.001** |  | **.09** | **<.001** |  | **.09** | **<.001** |  | **.06** | **.01** |  |
| Baseline maladjustment | **.37** | **<.001** |  | **.40** | **<.001** |  | **.31** | **<.001** |  | **.24** | **<.001** |  |

***Note***

SR = Self-report, PR=Parent-report, TR=Teacher-report, PeerR=Peer-report. Baseline maladjustment reflects equivalent measure to outcome self-reported at age 11. Coefficients with *p* < .05 are bolded.

***3c: Associations between peer victimization at age 13 and maladjustment at age 22***

|  | Withdrawal | | | Anxiety | | | Thought problems | | | Somatic complaints | | |
| --- | --- | --- | --- | --- | --- | --- | --- | --- | --- | --- | --- | --- |
|  | *ß* | *p* | *R^2^* | *ß* | *p* | *R^2^* | *ß* | *p* | *R^2^* | *ß* | *p* | *R^2^* |
| **Separate models per reporter** | | | | | | | | | | | | |
| SR peer victimization | -.004 | .88 | .11 | .03 | .33 | .16 | .05 | .05 | .10 | **.05** | **.03** | .17 |
| Sex | .04 | .08 |  | **-.10** | **<.001** |  | -.01 | .83 |  | **-.25** | **<.001** |  |
| Family SES | -.05 | .08 |  | .02 | .74 |  | **-.06** | **.03** |  | -.05 | .07 |  |
| Family structure | .04 | .11 |  | **.07** | **.01** |  | **.07** | **.01** |  | **.05** | **.04** |  |
| Baseline maladjustment | **.32** | **<.001** |  | **.35** | **<.001** |  | **.28** | **<.001** |  | **.25** | **<.001** |  |
| PR peer victimization | .05 | .09 | .11 | **.09** | **.001** | .17 | **.10** | **<.001** | .11 | **.10** | **<.001** | .18 |
| Sex | .04 | .12 |  | **-.10** | **<.001** |  | -.01 | .67 |  | **-.26** | **<.001** |  |
| Family SES | -.04 | .13 |  | .02 | .52 |  | -.05 | .05 |  | -.04 | .12 |  |
| Family structure | .04 | .12 |  | **.06** | **.01** |  | **.06** | **.02** |  | .04 | .08 |  |
| Baseline maladjustment | **.31** | **<.001** |  | **.33** | **<.001** |  | **.27** | **<.001** |  | **.25** | **<.001** |  |
| TR peer victimization | .05 | .07 | .11 | .05 | .08 | .16 | **.09** | **.001** | .11 | **.09** | **.002** | .18 |
| Sex | .04 | .14 |  | **-.10** | **<.001** |  | -.01 | .61 |  | **-.26** | **<.001** |  |
| Family SES | -.04 | .10 |  | .01 | .79 |  | **-.06** | **.02** |  | -.05 | .05 |  |
| Family structure | .04 | .11 |  | **.07** | **.01** |  | **.06** | **.02** |  | .05 | .06 |  |
| Baseline maladjustment | **.31** | **<.001** |  | **.35** | **<.001** |  | **.28** | **<.001** |  | **.25** | **<.001** |  |
| PeerR peer victimization | -.03 | .45 | .11 | -.02 | .64 | .16 | -.01 | .73 | .10 | -.01 | .79 | .17 |
| Sex | .05 | .06 |  | **-.09** | **<.001** |  | -.001 | .96 |  | **-.25** | **<.001** |  |
| Family SES | -.05 | .07 |  | .002 | .93 |  | **-.07** | **.01** |  | **-.05** | **.04** |  |
| Family structure | .05 | .09 |  | **.07** | **.01** |  | **.07** | **.01** |  | .05 | .05 |  |
| Baseline maladjustment | **.32** | **<.001** |  | **.36** | **<.001** |  | **.29** | **<.001** |  | **.26** | **<.001** |  |
|  | | | | | | | | | | | | |
| SR peer victimization | -.03 | .31 | .12 | -.01 | .72 | .17 | .001 | .98 | .11 | .004 | .89 | .18 |
| PR peer victimization | .04 | .19 |  | .08 | .01 |  | .08 | .01 |  | .08 | .01 |  |
| TR peer victimization | .07 | .05 |  | .04 | .24 |  | **.08** | **.02** |  | **.07** | **.03** |  |
| PeerR peer victimization | -.05 | .23 |  | -.05 | .27 |  | -.04 | .33 |  | -.05 | .25 |  |
| Sex | .04 | .11 |  | -.10 | <.001 |  | -.01 | .65 |  | **-.26** | **<.001** |  |
| Family SES | -.05 | .07 |  | .01 | .73 |  | **-.06** | **.03** |  | -.05 | .08 |  |
| Family structure | .04 | .11 |  | **.06** | **.01** |  | **.06** | **.02** |  | .05 | .06 |  |
| Baseline maladjustment | **.31** | **<.001** |  | **.34** | **<.001** |  | **.27** | **<.001** |  | **.25** | **<.001** |  |

***Note***

SR = Self-report, PR=Parent-report, TR=Teacher-report, PeerR=Peer-report. Baseline maladjustment reflects equivalent measure to outcome self-reported at age 11. Coefficients with *p* < .05 are bolded.

***3d: Associations between peer victimization at age 13 and maladjustment at age 26***

|  | Withdrawal | | | Anxiety | | | Thought problems | | | Somatic complaints | | |
| --- | --- | --- | --- | --- | --- | --- | --- | --- | --- | --- | --- | --- |
|  | *ß* | *p* | *R^2^* | *ß* | *p* | *R^2^* | *ß* | *p* | *R^2^* | *ß* | *p* | *R^2^* |
| **Separate models per reporter** | | | | | | | | | | | | |
| SR peer victimization | .03 | .35 | .10 | .02 | .55 | .14 | .04 | .14 | .09 | **.10** | **<.001** | .17 |
| Sex | **.08** | **.003** |  | **-.06** | **.02** |  | .04 | .11 |  | **-.21** | **<.001** |  |
| Family SES | **-.06** | **.03** |  | .03 | .36 |  | -.03 | .31 |  | -.05 | .08 |  |
| Family structure | **.10** | **<.001** |  | **.08** | **.004** |  | **.11** | **<.001** |  | .04 | .18 |  |
| Baseline maladjustment | **.28** | **<.001** |  | **.33** | **<.001** |  | **.26** | **<.001** |  | **.27** | **<.001** |  |
| PR peer victimization | **.09** | **.001** | .11 | **.10** | **<.001** | .15 | **.16** | **<.001** | .11 | **.14** | **<.001** | .18 |
| Sex | **.07** | **.01** |  | **-.07** | **.01** |  | .03 | .21 |  | **-.21** | **<.001** |  |
| Family SES | -.05 | .05 |  | .04 | .18 |  | -.01 | .66 |  | -.04 | .13 |  |
| Family structure | **.10** | **.001** |  | **.07** | **.01** |  | **.10** | **<.001** |  | .03 | .32 |  |
| Baseline maladjustment | **.26** | **<.001** |  | **.32** | **<.001** |  | **.25** | **<.001** |  | **.27** | **<.001** |  |
| TR peer victimization | **.07** | **.04** | .11 | .05 | .14 | .14 | **.09** | **.01** | .10 | **.12** | **<.001** | .17 |
| Sex | **.07** | **.01** |  | **-.07** | **.02** |  | .04 | .19 |  | **-.21** | **<.001** |  |
| Family SES | **-.06** | **.02** |  | .03 | .37 |  | -.03 | .27 |  | **-.06** | **.04** |  |
| Family structure | **.10** | **.001** |  | **.08** | **.01** |  | **.10** | **<.001** |  | .03 | .27 |  |
| Baseline maladjustment | **.27** | **<.001** |  | **.33** | **<.001** |  | **.26** | **<.001** |  | **.28** | **<.001** |  |
| PeerR peer victimization | .04 | .51 | .11 | .02 | .69 | .14 | .06 | .26 | .09 | .05 | .26 | .16 |
| Sex | **.08** | **.003** |  | **-.06** | **.02** |  | .04 | .13 |  | **-.20** | **<.001** |  |
| Family SES | **-.06** | **.03** |  | .03 | .36 |  | -.03 | .36 |  | -.05 | .06 |  |
| Family structure | **.10** | **.001** |  | **.08** | **.01** |  | **.10** | **<.001** |  | .03 | .27 |  |
| Baseline maladjustment | **.28** | **<.001** |  | **.34** | **<.001** |  | **.27** | **<.001** |  | **.28** | **<.001** |  |
| **Model with all reporters included simultaneously** | | | | | | | | | | | | |
| SR peer victimization | -.02 | .49 | .12 | -.03 | .29 | .15 | -.04 | .17 | .12 | .04 | .20 | .19 |
| PR peer victimization | **.08** | **.02** |  | **.09** | **.003** |  | **.15** | **<.001** |  | **.10** | **.001** |  |
| TR peer victimization | **.12** | **.001** |  | **.09** | **.01** |  | **.09** | **.01** |  | **.09** | **.01** |  |
| PeerR peer victimization | -.03 | .63 |  | -.03 | .59 |  | .02 | .65 |  | -.01 | .79 |  |
| Sex | **.07** | **.02** |  | **-.08** | **.01** |  | .02 | .39 |  | **-.22** | **<.001** |  |
| Family SES | -.04 | .13 |  | .04 | .13 |  | -.001 | .99 |  | -.03 | .29 |  |
| Family structure | **.09** | **.002** |  | **.07** | **.01** |  | **.09** | **.001** |  | .03 | .36 |  |
| Baseline maladjustment | **.26** | **<.001** |  | **.32** | **<.001** |  | **.25** | **<.001** |  | **.27** | **<.001** |  |

***Note***

SR = Self-report, PR=Parent-report, TR=Teacher-report, PeerR=Peer-report. Baseline maladjustment reflects equivalent measure to outcome self-reported at age 11. Coefficients with *p* < .05 are bolded.

***3e: Associations between peer victimization at age 13 and maladjustment at age 29***

|  | Withdrawal | | | Anxiety | | | Thought problems | | | Somatic complaints | | |
| --- | --- | --- | --- | --- | --- | --- | --- | --- | --- | --- | --- | --- |
|  | *ß* | *p* | *R^2^* | *ß* | *p* | *R^2^* | *ß* | *p* | *R^2^* | *ß* | *p* | *R^2^* |
| **Separate models per reporter** | | | | | | | | | | | | |
| SR peer victimization | .01 | .69 | .07 | .002 | .95 | .11 | .02 | .55 | .10 | .04 | .15 | .14 |
| Sex | **.07** | **.02** |  | -.06 | .05 |  | .04 | .22 |  | **-.16** | **<.001** |  |
| Family SES | -.06 | .06 |  | .001 | .99 |  | -.05 | .09 |  | -.04 | .18 |  |
| Family structure | **.09** | **.01** |  | **.09** | **.004** |  | .06 | .07 |  | .06 | .06 |  |
| Baseline maladjustment | **.23** | **<.001** |  | **.30** | **<.001** |  | **.29** | **<.001** |  | **.28** | **<.001** |  |
| PR peer victimization | **.08** | **.02** | .08 | **.09** | **.01** | .12 | **.10** | **.001** | .11 | **.09** | **.002** | .15 |
| Sex | **.06** | **.04** |  | **-.07** | **.02** |  | .03 | .33 |  | **-.17** | **<.001** |  |
| Family SES | -.05 | .11 |  | .01 | .69 |  | -.04 | .19 |  | -.04 | .26 |  |
| Family structure | **.08** | **.01** |  | **.08** | **.01** |  | .05 | .11 |  | .05 | .11 |  |
| Baseline maladjustment | **.22** | **<.001** |  | **.29** | **<.001** |  | **.28** | **<.001** |  | **.28** | **<.001** |  |
| TR peer victimization | .06 | .12 | .08 | **.07** | **.04** | .12 | **.08** | **.02** | .10 | **.09** | **.01** | .15 |
| Sex | **.06** | **.04** |  | **-.07** | **.02** |  | .03 | .34 |  | **-.17** | **<.001** |  |
| Family SES | -.06 | .06 |  | .004 | .90 |  | -.05 | .10 |  | -.04 | .17 |  |
| Family structure | **.08** | **.01** |  | **.09** | **.01** |  | .05 | .09 |  | .05 | .09 |  |
| Baseline maladjustment | **.23** | **<.001** |  | **.29** | **<.001** |  | **.29** | **<.001** |  | **.28** | **<.001** |  |
| PeerR peer victimization | -.03 | .57 | .07 | -.02 | .67 | .11 | .06 | .26 | .10 | .01 | .82 | .14 |
| Sex | **.07** | **.02** |  | -.06 | .07 |  | .03 | .29 |  | **-.16** | **<.001** |  |
| Family SES | **-.06** | **.04** |  | -.003 | .92 |  | -.05 | .13 |  | -.04 | .15 |  |
| Family structure | **.09** | **.01** |  | **.09** | **.004** |  | .05 | .11 |  | .05 | .07 |  |
| Baseline maladjustment | **.24** | **<.001** |  | **.30** | **<.001** |  | **.30** | **<.001** |  | **.29** | **<.001** |  |
| **Model with all reporters included simultaneously** | | | | | | | | | | | | |
| SR peer victimization | -.02 | .64 | .08 | -.03 | .33 | .13 | -.04 | .22 | .11 | -.001 | .97 | .15 |
| PR peer victimization | .07 | .05 |  | **.09** | **.01** |  | **.09** | **.01** |  | .07 | .05 |  |
| TR peer victimization | .05 | .17 |  | .06 | .10 |  | .05 | .17 |  | **.08** | **.03** |  |
| PeerR peer victimization | -.05 | .43 |  | -.06 | .27 |  | .05 | .37 |  | -.03 | .61 |  |
| Sex | **.06** | **.04** |  | **-.07** | **.03** |  | .02 | .47 |  | **-.17** | **<.001** |  |
| Family SES | -.06 | .08 |  | .003 | .92 |  | -.04 | .21 |  | -.04 | .23 |  |
| Family structure | **.08** | **.01** |  | **.08** | **.01** |  | .04 | .16 |  | .05 | .10 |  |
| Baseline maladjustment | **.22** | **<.001** |  | **.29** | **<.001** |  | **.29** | **<.001** |  | **.27** | **<.001** |  |

***Note***

SR = Self-report, PR=Parent-report, TR=Teacher-report, PeerR=Peer-report. Baseline maladjustment reflects equivalent measure to outcome self-reported at age 11. Coefficients with *p* < .05 are bolded.

**Supplementary Table 4**

***4a: Associations between chronic peer victimization and maladjustment at age 16***

|  | Withdrawal | | | Anxiety | | | Thought problems | | | Somatic complaints | | |
| --- | --- | --- | --- | --- | --- | --- | --- | --- | --- | --- | --- | --- |
|  | *ß* | *p* | *R^2^* | *ß* | *p* | *R^2^* | *ß* | *p* | *R^2^* | *ß* | *p* | *R^2^* |
| **Separate models per reporter** | | | | | | | | | | | | |
| SR chronic victimization | **.10** | **<.001** | .14 | **.06** | **.01** | .22 | **.07** | **.003** | .12 | **.09** | **<.001** | .21 |
| Sex | **-.12** | **<.001** |  | **-.28** | **<.001** |  | **-.08** | **<.001** |  | **-.31** | **<.001** |  |
| Family SES | -.05 | .05 |  | -.01 | .84 |  | -.02 | .37 |  | **-.07** | **.004** |  |
| Family structure | **.05** | **.03** |  | .03 | .26 |  | **.10** | **<.001** |  | .04 | .07 |  |
| Baseline maladjustment | **.30** | **<.001** |  | **.33** | **<.001** |  | **.30** | **<.001** |  | **.27** | **<.001** |  |
| PR chronic victimization | **.11** | **<.001** | .14 | **.11** | **<.001** | .22 | **.10** | **<.001** | .13 | **.12** | **<.001** | .22 |
| Sex | **-.12** | **<.001** |  | **-.29** | **<.001** |  | **-.09** | **<.001** |  | **-.32** | **<.001** |  |
| Family SES | **-.05** | **.04** |  | -.001 | .97 |  | -.02 | .37 |  | **-.07** | **.004** |  |
| Family structure | **.05** | **.03** |  | .03 | .27 |  | **.10** | **<.001** |  | .04 | .07 |  |
| Baseline maladjustment | **.30** | **<.001** |  | **.32** | **<.001** |  | **.31** | **<.001** |  | **.28** | **<.001** |  |
| TR chronic victimization | .04 | .22 | .13 | .02 | .58 | .21 | .04 | .27 | .12 | .03 | .32 | .21 |
| Sex | **-.12** | **<.001** |  | **-.28** | **<.001** |  | **-.09** | **<.001** |  | **-.31** | **<.001** |  |
| Family SES | **-.06** | **.01** |  | -.01 | .59 |  | -.03 | .20 |  | **-.08** | **.001** |  |
| Family structure | **.05** | **.03** |  | .03 | .23 |  | **.10** | **<.001** |  | .05 | .06 |  |
| Baseline maladjustment | **.32** | **<.001** |  | **.34** | **<.001** |  | **.31** | **<.001** |  | **.28** | **<.001** |  |
| PeerR chronic victimization | -.001 | .98 | .13 | .04 | .27 | .21 | -.03 | .44 | .12 | .06 | .11 | .21 |
| Sex | **-.11** | **<.001** |  | **-.29** | **<.001** |  | **-.08** | **.002** |  | **-.31** | **<.001** |  |
| Family SES | **-.06** | **.01** |  | -.01 | .59 |  | -.03 | .16 |  | **-.08** | **.001** |  |
| Family structure | **.05** | **.03** |  | .03 | .23 |  | **.10** | **<.001** |  | .05 | .05 |  |
| Baseline maladjustment | **.32** | **<.001** |  | **.33** | **<.001** |  | **.31** | **<.001** |  | **.28** | **<.001** |  |
| **Model with all reporters included simultaneously** | | | | | | | | | | | | |
| SR chronic victimization | **.07** | **.01** | .14 | .03 | .26 | .22 | .04 | .13 | .13 | **.06** | **.02** | .22 |
| PR chronic victimization | **.09** | **.001** |  | **.10** | **<.001** |  | **.09** | **.002** |  | **.08** | **.002** |  |
| TR chronic victimization | .01 | .67 |  | -.01 | .81 |  | .02 | .59 |  | -.01 | .69 |  |
| PeerR chronic victimization | -.02 | .69 |  | .03 | .42 |  | -.04 | .31 |  | .05 | .23 |  |
| Sex | **-.12** | **<.001** |  | **-.29** | **<.001** |  | **-.08** | **<.001** |  | **-.32** | **<.001** |  |
| Family SES | -.04 | .09 |  | .003 | .91 |  | -.02 | .51 |  | **-.06** | **.01** |  |
| Family structure | **.05** | **.04** |  | .02 | .29 |  | **.10** | **<.001** |  | .04 | .08 |  |
| Baseline maladjustment | **.29** | **<.001** |  | **.31** | **<.001** |  | **.30** | **<.001** |  | **.27** | **<.001** |  |

***Note***

SR = Self-report, PR=Parent-report, TR=Teacher-report, PeerR=Peer-report. Baseline maladjustment reflects equivalent measure to outcome self-reported at age 11. Coefficients with *p* < .05 are bolded.

***4b: Associations between chronic peer victimization and maladjustment at age 19***

|  | Withdrawal | | | Anxiety | | | Thought problems | | | Somatic complaints | | |
| --- | --- | --- | --- | --- | --- | --- | --- | --- | --- | --- | --- | --- |
|  | *ß* | *p* | *R^2^* | *ß* | *p* | *R^2^* | *ß* | *p* | *R^2^* | *ß* | *p* | *R^2^* |
| **Separate models per reporter** | | | | | | | | | | | | |
| SR chronic victimization | **.06** | **.03** | .09 | **.08** | **.001** | .16 | **.07** | **.01** | .09 | .03 | .25 | .11 |
| Sex | -.004 | .87 |  | **-.16** | **<.001** |  | -.02 | .41 |  | **-.22** | **<.001** |  |
| Family SES | -.05 | .07 |  | -.01 | .76 |  | -.03 | .31 |  | **-.08** | **.002** |  |
| Family structure | **.09** | **<.001** |  | **.10** | **<.001** |  | **.12** | **<.001** |  | **.07** | **.003** |  |
| Baseline maladjustment | **.25** | **<.001** |  | **.31** | **<.001** |  | **.25** | **<.001** |  | **.18** | **<.001** |  |
| PR chronic victimization | **.11** | **<.001** | .10 | **.15** | **<.001** | .17 | **.17** | **<.001** | .12 | **.13** | **<.001** | .12 |
| Sex | -.01 | .66 |  | **-.17** | **<.001** |  | -.03 | .18 |  | **-.23** | **<.001** |  |
| Family SES | -.04 | .11 |  | <.001 | .99 |  | -.02 | .52 |  | **-.07** | **.01** |  |
| Family structure | **.09** | **<.001** |  | **.09** | **<.001** |  | **.11** | **<.001** |  | **.06** | **.01** |  |
| Baseline maladjustment | **.24** | **<.001** |  | **.29** | **<.001** |  | **.25** | **<.001** |  | **.18** | **<.001** |  |
| TR chronic victimization | .05 | .15 | .09 | .04 | .18 | .15 | .06 | .07 | .09 | **.08** | **.01** | .11 |
| Sex | -.01 | .77 |  | **-.16** | **<.001** |  | -.02 | .33 |  | **-.23** | **<.001** |  |
| Family SES | -.05 | .05 |  | -.02 | .55 |  | -.03 | .20 |  | **-.08** | **.002** |  |
| Family structure | **.09** | **<.001** |  | **.10** | **<.001** |  | **.12** | **<.001** |  | **.07** | **.004** |  |
| Baseline maladjustment | **.26** | **<.001** |  | **.32** | **<.001** |  | **.25** | **<.001** |  | **.18** | **<.001** |  |
| PeerR chronic victimization | -.02 | .73 | .09 | .02 | .58 | .15 | .03 | .50 | .09 | .04 | .39 | .11 |
| Sex | .002 | .95 |  | **-.16** | **<.001** |  | -.02 | .41 |  | **-.22** | **<.001** |  |
| Family SES | **-.05** | **.04** |  | -.02 | .50 |  | -.05 | .17 |  | **-.08** | **.001** |  |
| Family structure | **.09** | **<.001** |  | **.10** | **<.001** |  | **.12** | **<.001** |  | **.07** | **.003** |  |
| Baseline maladjustment | **.26** | **<.001** |  | **.32** | **<.001** |  | **.25** | **<.001** |  | **.19** | **<.001** |  |
| **Model with all reporters included simultaneously** | | | | | | | | | | | | |
| SR chronic victimization | .02 | .38 | .10 | .03 | .25 | .17 | .01 | .76 | .12 | -.03 | .37 | .12 |
| PR chronic victimization | **.09** | **.001** |  | **.14** | **<.001** |  | **.15** | **<.001** |  | **.11** | **<.001** |  |
| TR chronic victimization | .03 | .37 |  | .02 | .64 |  | .04 | .222 |  | **.07** | **.043** |  |
| PeerR chronic victimization | -.03 | .46 |  | .003 | .95 |  | .01 | .853 |  | .02 | .635 |  |
| Sex | -.01 | .70 |  | **-.17** | **<.001** |  | -.04 | .134 |  | **-.24** | **<.001** |  |
| Family SES | -.04 | .13 |  | .003 | .91 |  | -.01 | .579 |  | **-.07** | **.006** |  |
| Family structure | **.09** | **<.001** |  | **.09** | **<.001** |  | **.11** | **<.001** |  | **.06** | **.008** |  |
| Baseline maladjustment | **.24** | **<.001** |  | **.29** | **<.001** |  | **.24** | **<.001** |  | **.18** | **<.001** |  |

***Note***

SR = Self-report, PR=Parent-report, TR=Teacher-report, PeerR=Peer-report. Baseline maladjustment reflects equivalent measure to outcome self-reported at age 11. Coefficients with *p* < .05 are bolded.

***4c: Associations between chronic peer victimization and maladjustment at age 22***

|  | Withdrawal | | | Anxiety | | | Thought problems | | | Somatic complaints | | |
| --- | --- | --- | --- | --- | --- | --- | --- | --- | --- | --- | --- | --- |
|  | *ß* | *p* | *R^2^* | *ß* | *p* | *R^2^* | *ß* | *p* | *R^2^* | *ß* | *p* | *R^2^* |
| **Separate models per reporter** | | | | | | | | | | | | |
| SR chronic victimization | .05 | .08 | .06 | **.08** | **.001** | .10 | **.06** | **.02** | .06 | **.05** | **.03** | .15 |
| Sex | .01 | .83 |  | **-.16** | **<.001** |  | -.04 | .16 |  | **-.29** | **<.001** |  |
| Family SES | -.04 | .12 |  | .02 | .40 |  | -.05 | .06 |  | **-.07** | **.01** |  |
| Family structure | .05 | .05 |  | **.06** | **.02** |  | **.08** | **.003** |  | .05 | .07 |  |
| Baseline maladjustment | **.22** | **<.001** |  | **.24** | **<.001** |  | **.20** | **<.001** |  | **.19** | **<.001** |  |
| PR chronic victimization | .03 | .31 | .06 | **.07** | **.01** | .10 | **.11** | **<.001** | .07 | **.09** | **.001** | .15 |
| Sex | .01 | .82 |  | **-.16** | **<.001** |  | -.04 | .10 |  | **-.30** | **<.001** |  |
| Family SES | -.05 | .10 |  | .02 | .46 |  | -.05 | .08 |  | **-.07** | **.01** |  |
| Family structure | .05 | .06 |  | **.06** | **.02** |  | **.08** | **.01** |  | .04 | .10 |  |
| Baseline maladjustment | **.22** | **<.001** |  | **.24** | **<.001** |  | **.20** | **<.001** |  | **.19** | **<.001** |  |
| TR chronic victimization | .02 | .59 | .06 | .01 | .74 | .10 | .06 | .11 | .06 | .04 | .23 | .14 |
| Sex | .01 | .82 |  | **-.16** | **<.001** |  | -.04 | .13 |  | **-.29** | **<.001** |  |
| Family SES | -.05 | .08 |  | .01 | .68 |  | **-.06** | **.03** |  | **-.07** | **.01** |  |
| Family structure | .05 | .06 |  | **.06** | **.02** |  | **.08** | **.003** |  | .05 | .08 |  |
| Baseline maladjustment | **.23** | **<.001** |  | **.25** | **<.001** |  | **.21** | **<.001** |  | **.19** | **<.001** |  |
| PeerR chronic victimization | -.07 | .12 | .07 | -.02 | .70 | .10 | -.07 | .14 | .07 | -.01 | .83 | .14 |
| Sex | .02 | .46 |  | **-.15** | **<.001** |  | -.02 | .42 |  | **-.29** | **<.001** |  |
| Family SES | -.05 | .06 |  | .01 | .71 |  | **-.06** | **.03** |  | **-.08** | **.003** |  |
| Family structure | .05 | .06 |  | **.06** | **.02** |  | **.08** | **.003** |  | .05 | .07 |  |
| Baseline maladjustment | **.24** | **<.001** |  | **.25** | **<.001** |  | **.21** | **<.001** |  | **.20** | **<.001** |  |
| **Model with all reporters included simultaneously** | | | | | | | | | | | | |
| SR chronic victimization | .04 | .19 | .07 | .07 | .02 | .11 | .02 | .41 | .08 | .02 | .40 | .15 |
| PR chronic victimization | .03 | .34 |  | .06 | .05 |  | **.10** | **<.001** |  | **.08** | **.01** |  |
| TR chronic victimization | .02 | .56 |  | -.01 | .89 |  | .05 | .18 |  | .03 | .43 |  |
| PeerR chronic victimization | -.08 | .09 |  | -.02 | .57 |  | -.09 | .06 |  | -.02 | .60 |  |
| Sex | .01 | .64 |  | **-.16** | **<.001** |  | -.04 | .17 |  | **-.30** | **<.001** |  |
| Family SES | -.04 | .14 |  | .03 | .33 |  | -.05 | .10 |  | **-.06** | **.02** |  |
| Family structure | .05 | .06 |  | **.06** | **.02** |  | **.08** | **.004** |  | .04 | .10 |  |
| Baseline maladjustment | **.22** | **<.001** |  | **.23** | **<.001** |  | **.20** | **<.001** |  | **.19** | **<.001** |  |

***Note***

SR = Self-report, PR=Parent-report, TR=Teacher-report, PeerR=Peer-report. Baseline maladjustment reflects equivalent measure to outcome self-reported at age 11. Coefficients with *p* < .05 are bolded.

***4d: Associations between chronic peer victimization and maladjustment at age 26***

|  | Withdrawal | | | Anxiety | | | Thought problems | | | Somatic complaints | | |
| --- | --- | --- | --- | --- | --- | --- | --- | --- | --- | --- | --- | --- |
|  | *ß* | *p* | *R^2^* | *ß* | *p* | *R^2^* | *ß* | *p* | *R^2^* | *ß* | *p* | *R^2^* |
| **Separate models per reporter** | | | | | | | | | | | | |
| SR chronic victimization | .04 | .20 | .08 | .04 | .18 | .09 | .01 | .83 | .06 | **.09** | **.002** | .11 |
| Sex | .05 | .06 |  | **-.11** | **<.001** |  | .02 | .47 |  | **-.26** | **<.001** |  |
| Family SES | **-.06** | **.03** |  | .03 | .23 |  | -.03 | .30 |  | **-.07** | **.02** |  |
| Family structure | **.10** | **<.001** |  | **.08** | **.01** |  | **.12** | **<.001** |  | .03 | .30 |  |
| Baseline maladjustment | **.23** | **<.001** |  | **.25** | **<.001** |  | **.21** | **<.001** |  | **.15** | **<.001** |  |
| PR chronic victimization | **.08** | **.01** | .09 | **.10** | **.001** | .10 | **.12** | **<.001** | .08 | **.13** | **<.001** | .12 |
| Sex | .05 | .09 |  | **-.12** | **<.001** |  | .01 | .76 |  | **-.26** | **<.001** |  |
| Family SES | -.06 | .05 |  | .04 | .13 |  | -.02 | .59 |  | **-.06** | **.03** |  |
| Family structure | **.10** | **<.001** |  | **.07** | **.01** |  | **.11** | **<.001** |  | .02 | .39 |  |
| Baseline maladjustment | **.22** | **<.001** |  | **.23** | **<.001** |  | **.20** | **<.001** |  | **.15** | **<.001** |  |
| TR chronic victimization | .04 | .28 | .08 | .02 | .61 | .09 | .05 | .21 | .07 | .06 | .11 | .11 |
| Sex | .05 | .09 |  | **-.11** | **<.001** |  | .01 | .65 |  | **-.26** | **<.001** |  |
| Family SES | **-.06** | **.03** |  | .03 | .28 |  | -.03 | .33 |  | **-.08** | **.01** |  |
| Family structure | **.10** | **<.001** |  | **.08** | **.01** |  | **.12** | **<.001** |  | .03 | .28 |  |
| Baseline maladjustment | **.23** | **<.001** |  | **.25** | **<.001** |  | **.20** | **<.001** |  | **.15** | **<.001** |  |
| PeerR chronic victimization | -.03 | .51 | .08 | -.02 | .74 | .09 | .03 | .52 | .07 | .03 | .50 | .10 |
| Sex | **.06** | **.04** |  | **-.11** | **<.001** |  | .02 | .60 |  | **-.25** | **<.001** |  |
| Family SES | **-.07** | **.02** |  | .03 | .30 |  | -.03 | .30 |  | **-.08** | **.01** |  |
| Family structure | **.10** | **<.001** |  | **.08** | **.01** |  | **.12** | **<.001** |  | .03 | .26 |  |
| Baseline maladjustment | **.24** | **<.001** |  | **.25** | **<.001** |  | **.21** | **<.001** |  | **.16** | **<.001** |  |
| **Model with all reporters included simultaneously** | | | | | | | | | | | | |
| SR chronic victimization | .008 | .80 | .09 | .01 | .80 | .10 | -.05 | .15 | .08 | .04 | .16 | .13 |
| PR chronic victimization | **.076** | **.02** |  | **.09** | **.003** |  | **.11** | **<.001** |  | **.10** | **.001** |  |
| TR chronic victimization | .046 | .26 |  | .01 | .72 |  | .05 | .21 |  | .05 | .21 |  |
| PeerR chronic peer victimization | -.044 | .39 |  | -.03 | .64 |  | .02 | .70 |  | .02 | .75 |  |
| Sex | .045 | .12 |  | **-.12** | **<.001** |  | <.001 | .99 |  | **-.27** | **<.001** |  |
| Family SES | -.053 | .06 |  | .04 | .14 |  | -.02 | .55 |  | -.06 | .05 |  |
| Family structure | **.099** | **<.001** |  | **.07** | **.01** |  | **.12** | **<.001** |  | .02 | .39 |  |
| Baseline maladjustment | **.219** | **<.001** |  | **.23** | **<.001** |  | **.20** | **<.001** |  | **.15** | **<.001** |  |

***Note***

SR = Self-report, PR=Parent-report, TR=Teacher-report, PeerR=Peer-report. Baseline maladjustment reflects equivalent measure to outcome self-reported at age 11. Coefficients with *p* < .05 are bolded.

***4e: Associations between chronic peer victimization and maladjustment at age 29***

|  | Withdrawal | | | Anxiety | | | Thought problems | | | Somatic complaints | | |
| --- | --- | --- | --- | --- | --- | --- | --- | --- | --- | --- | --- | --- |
|  | *ß* | *p* | *R^2^* | *ß* | *p* | *R^2^* | *ß* | *p* | *R^2^* | *ß* | *p* | *R^2^* |
| **Separate models per reporter** | | | | | | | | | | | | |
| SR chronic victimization | .02 | .48 | .07 | **.07** | **.03** | .08 | .05 | .14 | .06 | .05 | .14 | .09 |
| Sex | .05 | .13 |  | **-.11** | **<.001** |  | .004 | .89 |  | **-.21** | **<.001** |  |
| Family SES | **-.07** | **.04** |  | .01 | .75 |  | -.05 | .14 |  | **-.07** | **.03** |  |
| Family structure | **.08** | **.01** |  | **.08** | **.01** |  | **.07** | **.03** |  | .05 | .09 |  |
| Baseline maladjustment | **.22** | **<.001** |  | **.22** | **<.001** |  | **.20** | **<.001** |  | **.16** | **<.001** |  |
| PR chronic victimization | .02 | .63 | .07 | .04 | .226 | .08 | .06 | .09 | .06 | **.09** | **.01** | .10 |
| Sex | .05 | .13 |  | **-.11** | **<.001** |  | .003 | .93 |  | **-.22** | **<.001** |  |
| Family SES | **-.07** | **.04** |  | .01 | .87 |  | -.05 | .15 |  | **-.06** | **.04** |  |
| Family structure | **.08** | **.01** |  | **.08** | **.01** |  | **.07** | **.03** |  | .05 | .12 |  |
| Baseline maladjustment | **.22** | **<.001** |  | **.23** | **<.001** |  | **.20** | **<.001** |  | **.16** | **<.001** |  |
| TR chronic victimization | .04 | .40 | .07 | -.02 | .64 | .08 | .04 | .42 | .06 | .07 | .10 | .09 |
| Sex | .04 | .17 |  | **-.10** | **.001** |  | .004 | .90 |  | **-.22** | **<.001** |  |
| Family SES | **-.07** | **.04** |  | -.001 | .97 |  | -.05 | .11 |  | **-.07** | **.02** |  |
| Family structure | **.08** | **.01** |  | **.08** | **.01** |  | **.07** | **.03** |  | .05 | .10 |  |
| Baseline maladjustment | **.22** | **<.001** |  | **.23** | **<.001** |  | **.20** | **<.001** |  | **.16** | **<.001** |  |
| PeerR chronic victimization | -.04 | .51 | .07 | -.07 | .21 | .08 | .08 | .16 | .06 | .01 | .93 | .09 |
| Sex | .05 | .09 |  | **-.09** | **.004** |  | -.004 | .90 |  | **-.21** | **<.001** |  |
| Family SES | **-.07** | **.03** |  | -.002 | .95 |  | -.05 | .11 |  | **-.08** | **.01** |  |
| Family structure | **.08** | **.01** |  | **.08** | **.01** |  | **.07** | **.02** |  | .05 | .08 |  |
| Baseline maladjustment | **.23** | **<.001** |  | **.24** | **<.001** |  | **.20** | **<.001** |  | **.16** | **<.001** |  |
| **Model with all reporters included simultaneously** | | | | | | | | | | | | |
| SR chronic victimization | .01 | .69 | .08 | **.07** | **.04** | .09 | .03 | .43 | .06 | .01 | .66 | .10 |
| PR chronic victimization | .01 | .77 |  | .04 | .25 |  | .03 | .37 |  | **.07** | **.04** |  |
| TR chronic victimization | .04 | .40 |  | -.04 | .42 |  | .02 | .72 |  | .06 | .19 |  |
| PeerR chronic peer victimization | -.04 | .41 |  | -.08 | .15 |  | .07 | .20 |  | -.01 | .81 |  |
| Sex | .05 | .14 |  | **-.10** | **.002** |  | -.01 | .70 |  | **-.22** | **<.001** |  |
| Family SES | -.06 | .05 |  | .01 | .75 |  | -.04 | .20 |  | -.06 | .05 |  |
| Family structure | **.08** | **.01** |  | **.08** | **.01** |  | **.07** | **.03** |  | .05 | .14 |  |
| Baseline maladjustment | **.22** | **<.001** |  | **.22** | **<.001** |  | **.19** | **<.001** |  | **.16** | **<.001** |  |

***Note***

SR = Self-report, PR=Parent-report, TR=Teacher-report, PeerR=Peer-report. Baseline maladjustment reflects equivalent measure to outcome self-reported at age 11. Coefficients with *p* < .05 are bolded.

**Supplementary Table 5**

***5a: Associations between peer victimization at age 11 and maladjustment as latent construct at age 13***

|  | Maladjustment | | |
| --- | --- | --- | --- |
|  | *ß* | *p* | *R^2^* |
| **Separate models per reporter** |  |  |  |
| SR peer victimization | .01 | .55 | .41 |
| Sex | **-.23** | **<.001** |  |
| Family SES | .01 | .70 |  |
| Family structure | .02 | .25 |  |
| Baseline maladjustment | **.57** | **<.001** |  |
| PR peer victimization | **.11** | **<.001** | .42 |
| Sex | **-.24** | **<.001** |  |
| Family SES | .02 | .34 |  |
| Family structure | .02 | .35 |  |
| Baseline maladjustment | **.552** | **<.001** |  |
| TR peer victimization | .042 | .07 | .41 |
| Sex | **-.23** | **<.001** |  |
| Family SES | .01 | .56 |  |
| Family structure | .02 | .28 |  |
| Baseline maladjustment | **.57** | **<.001** |  |
| PeerR peer victimization | .04 | .15 | .42 |
| Sex | **-.23** | **<.001** |  |
| Family SES | .01 | .64 |  |
| Family structure | .02 | .28 |  |
| Baseline maladjustment | **.58** | **<.001** |  |
| **Model with all reporters included simultaneously** |  |  |  |
| SR peer victimization | -.04 | .15 | .42 |
| PR peer victimization | **.11** | **<.001** |  |
| TR peer victimization | .01 | .75 |  |
| PeerR peer victimization | .03 | .30 |  |
| Sex | **-.24** | **<.001** |  |
| Family SES | .02 | .30 |  |
| Family structure | .02 | .38 |  |
| Baseline maladjustment | **.57** | **<.001** |  |

***Note***

SR = Self-report, PR=Parent-report, TR=Teacher-report, PeerR=Peer-report. Baseline maladjustment reflects equivalent measure to outcome self-reported at age 11. Coefficients with *p* < .05 are bolded.

***5b: Associations between peer victimization at age 11 and maladjustment as latent construct at age 16***

|  | Maladjustment | | |
| --- | --- | --- | --- |
|  | *ß* | *p* | *R^2^* |
| **Separate models per reporter** |  |  |  |
| SR peer victimization | -.01 | .85 | .30 |
| Sex | **-.27** | **<.001** |  |
| Family SES | -.04 | .08 |  |
| Family structure | .05 | .05 |  |
| Baseline maladjustment | **.44** | **<.001** |  |
| PR peer victimization | **.10** | **<.001** | .31 |
| Sex | **-.28** | **<.001** |  |
| Family SES | -.03 | .28 |  |
| Family structure | .04 | .07 |  |
| Baseline maladjustment | **.41** | **<.001** |  |
| TR peer victimization | .05 | .05 | .30 |
| Sex | **-.28** | **<.001** |  |
| Family SES | -.04 | .17 |  |
| Family structure | .05 | .06 |  |
| Baseline maladjustment | **.43** | **<.001** |  |
| PeerR peer victimization | -.03 | .44 | .30 |
| Sex | **-.27** | **<.001** |  |
| Family SES | -.05 | .07 |  |
| Family structure | .05 | .04 |  |
| Baseline maladjustment | **.44** | **<.001** |  |
| **Model with all reporters included simultaneously** |  |  |  |
| SR peer victimization | -.05 | .09 | .31 |
| PR peer victimization | **.12** | **<.001** |  |
| TR peer victimization | .03 | .27 |  |
| PeerR peer victimization | -.03 | .43 |  |
| Sex | **-.28** | **<.001** |  |
| Family SES | -.03 | .26 |  |
| Family structure | .04 | .08 |  |
| Baseline maladjustment | **.43** | **<.001** |  |

***Note***

SR = Self-report, PR=Parent-report, TR=Teacher-report, PeerR=Peer-report. Baseline maladjustment reflects equivalent measure to outcome self-reported at age 11. Coefficients with *p* < .05 are bolded.

***5c: Associations between peer victimization at age 11 and maladjustment as latent construct at age 19***

|  | Maladjustment | | |
| --- | --- | --- | --- |
|  | *ß* | *p* | *R^2^* |
| **Separate models per reporter** |  |  |  |
| SR peer victimization | .01 | .64 | .21 |
| Sex | **-.13** | **<.001** |  |
| Family SES | -.03 | .21 |  |
| Family structure | **.11** | **<.001** |  |
| Baseline maladjustment | **.40** | **<.001** |  |
| PR peer victimization | **.16** | **<.001** | .24 |
| Sex | **-.14** | **<.001** |  |
| Family SES | -.01 | .65 |  |
| Family structure | **.10** | **<.001** |  |
| Baseline maladjustment | **.37** | **<.001** |  |
| TR peer victimization | **.13** | **<.001** | .23 |
| Sex | **-.14** | **<.001** |  |
| Family SES | -.02 | .53 |  |
| Family structure | **.10** | **<.001** |  |
| Baseline maladjustment | **.38** | **<.001** |  |
| PeerR peer victimization | -.03 | .42 | .21 |
| Sex | **-.12** | **<.001** |  |
| Family SES | -.04 | .15 |  |
| Family structure | **.11** | **<.001** |  |
| Baseline maladjustment | **.41** | **<.001** |  |
| **Model with all reporters included simultaneously** |  |  |  |
| SR peer victimization | -.06 | .06 | .25 |
| PR peer victimization | **.17** | **<.001** |  |
| TR peer victimization | **.11** | **<.001** |  |
| PeerR peer victimization | -.07 | .08 |  |
| Sex | **-.14** | **<.001** |  |
| Family SES | -.01 | .76 |  |
| Family structure | **.10** | **<.001** |  |
| Baseline maladjustment | **.39** | **<.001** |  |

***Note***

SR = Self-report, PR=Parent-report, TR=Teacher-report, PeerR=Peer-report. Baseline maladjustment reflects equivalent measure to outcome self-reported at age 11. Coefficients with *p* < .05 are bolded.

***5d: Associations between peer victimization at age 11 and maladjustment as latent construct at age 22***

|  | Maladjustment | | |
| --- | --- | --- | --- |
|  | *ß* | *p* | *R^2^* |
| **Separate models per reporter** |  |  |  |
| SR peer victimization | -.003 | .93 | .14 |
| Sex | **-.14** | **<.001** |  |
| Family SES | -.03 | .39 |  |
| Family structure | **.07** | **.02** |  |
| Baseline maladjustment | **.33** | **<.001** |  |
| PR peer victimization | **.11** | **<.001** | .15 |
| Sex | **-.15** | **<.001** |  |
| Family SES | -.01 | .77 |  |
| Family structure | **.06** | **.02** |  |
| Baseline maladjustment | **.30** | **<.001** |  |
| TR peer victimization | **.07** | **.03** | .15 |
| Sex | **-.15** | **<.001** |  |
| Family SES | -.01 | .65 |  |
| Family structure | **.07** | **.01** |  |
| Baseline maladjustment | **.31** | **<.001** |  |
| PeerR peer victimization | -.02 | .65 | .14 |
| Sex | **-.14** | **<.001** |  |
| Family SES | -.03 | .34 |  |
| Family structure | **.07** | **.01** |  |
| Baseline maladjustment | **.33** | **<.001** |  |
| **Model with all reporters included simultaneously** |  |  |  |
| SR peer victimization | -.05 | .16 | .16 |
| PR peer victimization | **.12** | **<.001** |  |
| TR peer victimization | .06 | .10 |  |
| PeerR peer victimization | -.05 | .27 |  |
| Sex | **-.15** | **<.001** |  |
| Family SES | -.01 | .79 |  |
| Family structure | **.06** | **.03** |  |
| Baseline maladjustment | **.31** | **<.001** |  |

***Note***

SR = Self-report, PR=Parent-report, TR=Teacher-report, PeerR=Peer-report. Baseline maladjustment reflects equivalent measure to outcome self-reported at age 11. Coefficients with *p* < .05 are bolded.

***5e: Associations between peer victimization at age 11 and maladjustment as latent construct at age 26***

|  | Maladjustment | | |
| --- | --- | --- | --- |
|  | *ß* | *p* | *R^2^* |
| **Separate models per reporter** |  |  |  |
| SR peer victimization | -.02 | .58 | .13 |
| Sex | **-.08** | **.01** |  |
| Family SES | -.02 | .57 |  |
| Family structure | **.10** | **.001** |  |
| Baseline maladjustment | **.35** | **<.001** |  |
| PR peer victimization | **.15** | **<.001** | .13 |
| Sex | **-.09** | **.001** |  |
| Family SES | .01 | .79 |  |
| Family structure | **.08** | **.01** |  |
| Baseline maladjustment | **.30** | **<.001** |  |
| TR peer victimization | **.11** | **.001** | .13 |
| Sex | **-.09** | **.001** |  |
| Family SES | .00 | .89 |  |
| Family structure | **.09** | **.003** |  |
| Baseline maladjustment | **.32** | **<.001** |  |
| PeerR peer victimization | .004 | .92 | .13 |
| Sex | **-.08** | **.01** |  |
| Family SES | -.02 | .61 |  |
| Family structure | **.10** | **.001** |  |
| Baseline maladjustment | **.34** | **<.001** |  |
| **Model with all reporters included simultaneously** |  |  |  |
| SR peer victimization | **-.10** | **.01** | .14 |
| PR peer victimization | **.16** | **<.001** |  |
| TR peer victimization | **.10** | **.01** |  |
| PeerR peer victimization | -.02 | .61 |  |
| Sex | **-.10** | **.001** |  |
| Family SES | .01 | .74 |  |
| Family structure | **.08** | **.01** |  |
| Baseline maladjustment | **.33** | **<.001** |  |

***Note***

SR = Self-report, PR=Parent-report, TR=Teacher-report, PeerR=Peer-report. Baseline maladjustment reflects equivalent measure to outcome self-reported at age 11. Coefficients with *p* < .05 are bolded.

***5f: Associations between peer victimization at age 11 and maladjustment as latent construct at age 29***

|  | Maladjustment | | |
| --- | --- | --- | --- |
|  | *ß* | *p* | *R^2^* |
| **Separate models per reporter** |  |  |  |
| SR peer victimization | -.02 | .67 | .13 |
| Sex | **-.07** | **.03** |  |
| Family SES | -.04 | .19 |  |
| Family structure | **.08** | **.01** |  |
| Baseline maladjustment | **.32** | **<.001** |  |
| PR peer victimization | **.08** | **.03** | .13 |
| Sex | **-.08** | **.01** |  |
| Family SES | -.03 | .40 |  |
| Family structure | **.08** | **.02** |  |
| Baseline maladjustment | **.30** | **<.001** |  |
| TR peer victimization | **.08** | **.02** | .13 |
| Sex | **-.08** | **.01** |  |
| Family SES | -.09 | .39 |  |
| Family structure | **.08** | **.02** |  |
| Baseline maladjustment | **.30** | **<.001** |  |
| PeerR peer victimization | .01 | .89 | .13 |
| Sex | **-.07** | **.03** |  |
| Family SES | -.04 | .23 |  |
| Family structure | **.09** | **.01** |  |
| Baseline maladjustment | **.32** | **<.001** |  |
| **Model with all reporters included simultaneously** |  |  |  |
| SR peer victimization | -.07 | .09 | .14 |
| PR peer victimization | **.09** | **.02** |  |
| TR peer victimization | .07 | .09 |  |
| PeerR peer victimization | -.01 | .89 |  |
| Sex | **-.08** | **.01** |  |
| Family SES | -.03 | .44 |  |
| Family structure | **.07** | **.02** |  |
| Baseline maladjustment | **.32** | **<.001** |  |

***Note***

SR = Self-report, PR=Parent-report, TR=Teacher-report, PeerR=Peer-report. Baseline maladjustment reflects equivalent measure to outcome self-reported at age 11. Coefficients with *p* < .05 are bolded.

Supplementary Figure 1: Heatmap depicting pairwise correlations between study variables.


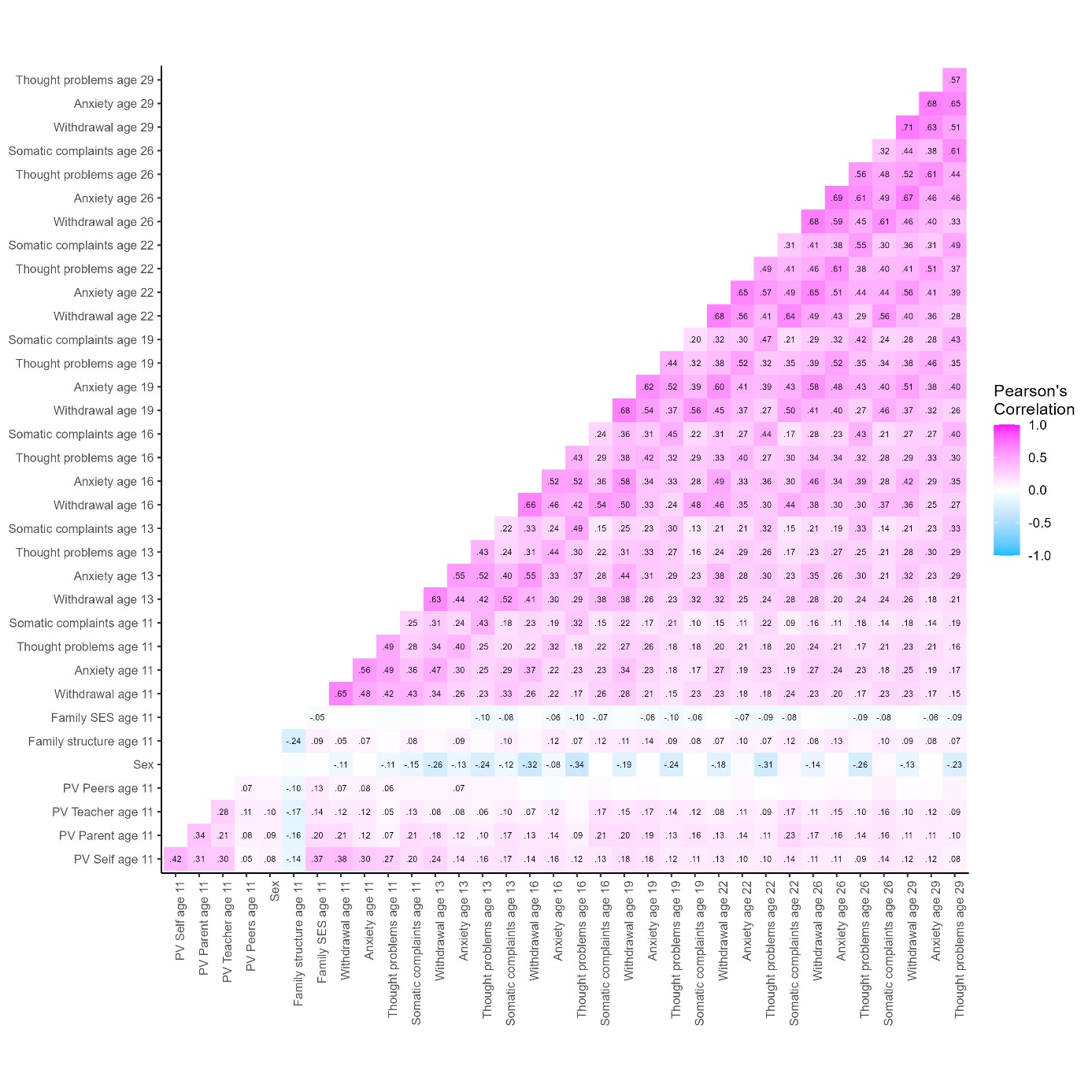


***Note***

PV=Peer Victimization. Only associations with p<.05 are depicted.
